# The Relational Nature of Attachment and Power: Attachment Avoidance and Withdrawal Limit Partners’ Power

**DOI:** 10.1177/01461672251333165

**Published:** 2025-04-29

**Authors:** Robert Körner, Nickola C. Overall, Valerie T. Chang, Matthew D. Hammond, Eri Sasaki, Astrid Schütz, Erez Zverling

**Affiliations:** 1 University of Bamberg, Germany; 2 University of Auckland, New Zealand; 3 Victoria University of Wellington, New Zealand; 4 University of Toronto, ON, Canada; 5 College of Law and Business, Ramat Gan, Israel; 6 University of Haifa, Israel

**Keywords:** power, attachment, APIM, romantic relationships, withdrawal

## Abstract

Power emerges from the relational dynamics between two people, but it is often studied as a feature of the individual. The current studies apply a dyadic perspective to show that core relational beliefs not only shape actors’ relationship power but also generate behaviors that constrain their partner’s power. Across five studies with Israeli, German, and New Zealand couples (total 1,256 dyads), greater attachment avoidance and anxiety were associated with lower actor power. Revealing novel dyadic effects, greater attachment avoidance was associated with partner’s experiencing lower power. Studies 4 and 5 showed that actors higher in avoidance enacted greater withdrawal during conflict and daily life as perceived by partners and observed by independent coders. Actors’ withdrawal, in turn, predicted partners experiencing lower power. These findings advance power and relationship theories and research by highlighting how relational characteristics and behaviors (withdrawal) likely shape the experience of both actors’ and partners’ power in social relationships.

Attachment and power are both fundamental components of social relationships (Agnew & Harman, 2019; Mikulincer & Shaver, 2003). Yet, how and why attachment orientations may shape the power held by both people within close relationships is poorly understood. Understanding these links requires a dyadic approach that recognizes how actors and partners influence each other. Relationship power reflects an actor’s ability to influence their partner to achieve desired outcomes (Anderson et al., 2012; Simpson et al., 2015). Critically, in interdependent relationships, actors are also subject to their partner’s power to influence them (Overall et al., 2023; Simpson et al., 2015; Thibaut & Kelley, 1978). Early assessments of relative power (i.e., who possesses more power) were ambiguous about whether the predictors and consequences of power were related to actors lacking power or partners possessing power. By contrast, growing evidence shows that actor and partner power are distinct and have differential effects in relationships. Actors low in power inhibit their own needs and goals and thus experience lower relationship satisfaction, whereas actors high in power can confidently express and pursue their own needs and goals and thus experience higher relationship satisfaction (Körner & Schütz, 2021; Overall et al., 2023). Actor’s behavior is also independently determined by their partner’s power: actors are motivated to accommodate the needs and goals of partners who have high power, whereas they may neglect the needs of partners who have low power (Körner et al., 2022; Overall et al., 2023).

Understanding the way attachment insecurity shapes relationships also requires a dyadic approach that recognizes how actors and partners influence each other. Actors’ own attachment insecurity is associated with destructive behavior that damages actors’ and partners’ relationship quality (McNulty et al., 2021; Overall et al., 2022). Partners’ attachment insecurity also independently affects the way actors behave and evaluate their relationship (McNulty et al., 2021; Overall et al., 2022). As outlined below, these attachment dynamics arise from behavioral strategies designed to manage dependence and power in relationships, yet no prior studies have systematically examined how attachment insecurity relates to the way both actors and partners experience power. Instead, prior theory and research has primarily focused on sociodemographic or societal (e.g., majority group status) characteristics as antecedents of power (e.g., Keltner et al., 2003; Vogler et al., 2008), overlooking that people’s experience of relationship power is distinct from, and has stronger effects on relationship outcomes than objective sources of power (e.g., socioeconomic status; Anderson et al., 2012; Körner & Schütz, 2021; Weisfeld et al., 1992). In the current research, we illustrate the importance of a dyadic approach to examine how core *relational* attributes that involve managing dependence and power in relationships (attachment insecurity) are related to both *actors’* and *partners’* relationship power. We first present three studies providing the first direct tests of the associations between actors’ and partners’ attachment insecurity and power. We then present two studies testing the behavioral strategies (withdrawal) that help explain why actors’ attachment avoidance is associated with *partners*’ lower power.

## Attachment Avoidance and Power Within Close Relationships

Attachment avoidance is theorized to arise when people have experienced neglect within important relationships, producing beliefs that close others cannot be relied upon when needed (Bowlby, 1973). Such distrust and expectations that partners will be unresponsive result in a reluctance for closeness and preference for maintaining independence instead of depending on partners for care and support (Mikulincer & Shaver, 2003). For this reason, avoidant individuals engage in distancing and withdrawal to reduce dependence and sustain control—that is, to maintain a sense of power (Overall, 2019; Overall & Cross, 2019). Individuals higher in avoidance routinely exhibit lower disclosure (Tan et al., 2012) and support seeking (Collins & Feeney, 2000) and greater withdrawal (Pietromonaco & Feldman Barrett, 1997). Such withdrawal is particularly evident when avoidant individuals are vulnerable and their power is challenged, including during stressful events (Fraley & Shaver, 1998; Simpson et al., 1992), when couples discuss conflict (Barry & Lawrence, 2013; McNulty et al., 2021; Simpson et al., 1996), and when subject to their partners’ influence attempts (Overall & Sibley, 2009; Overall et al., 2013).

The specific needs, goals, and behavioral strategies associated with attachment avoidance should shape the experience of relationship power (see Table 1). The distancing and withdrawal described above aim to limit dependence and sustain control and, thus, may be assumed to help people high in avoidance retain a sense of power. For example, people high in attachment avoidance are less committed to their relationship (Simpson, 1990), which may make them feel less affected by what their partner does, thereby protecting their sense of power (Sprecher et al., 2006). However, the constant need to restore control clashes with interdependence (Overall, 2019). Strong desires to protect or retain power are difficult to meet in close relationships because partners inevitably impact the attainment of needs and goals to some degree (Simpson et al., 2015; Thibaut & Kelly, 1978). Faced with this reality, avoidant individuals are likely to experience any constraints to power as particularly salient and significant, resulting in experiencing lower power. Prior research has shown, for example, that people who are particularly concerned with retaining power perceive that they have less influence than their partners actually report being influenced by them (Cross et al., 2019). Avoidant individuals also show power-relevant biases in their perceptions of couple interactions, such as interpreting their partner’s need for support as controlling or manipulative (Simpson et al., 2002; Girme et al., 2015), impinging on their power. Additionally, avoidant distancing strategies stem from beliefs that relying on partners leaves them vulnerable to lack of care and neglect and, thus, a belief that they are unable to safely obtain their needs and goals (Mikulincer & Shaver, 2003). These underlying threat-based conditions represent a perceived lack of power rather than a sense of security and ability to control desired outcomes (Keltner et al., 2003). Thus, actors high in avoidance are likely to experience lower relationship power.

**Table 1.** Summary of Attachment Dynamics That Lead to Predictions Regarding Links Between Attachment Avoidance and Anxiety and Actors’ and Partners’ Experience of Power.

Attachment dimension	Actors’ experience of power	Partners’ experience of power
Attachment avoidance	*Attachment dynamics*: Mistrust of partners lead to prioritizing independence and distancing from partner to reduce dependence and restore control Partner’s dependence and influence is interpreted as controlling and impedes need for control *Prediction*: Higher attachment avoidance will be associated with actors experiencing low power	*Attachment dynamics*: Behavioral strategies to restore control (withdrawal) limits partner’s power, conveys actors’ low investment, and may force partners to soften their influence *Prediction*: Higher attachment avoidance will be associated with partners experiencing low power
Attachment anxiety	Concern for closeness and fear of rejection amplifies dependence on the partner Constant reassurance seeking and behaviors designed to obtain love and care *Prediction*: Higher attachment anxiety will be associated with actors experiencing low power	High needs for acceptance and closeness may *increase* partner’s power as they have control over valued resource Anxious individuals’ behavioral strategies may *reduce* partner’s power by eliciting partners’ reassurance and expressed dependence *Prediction*: Mixed or null associations between attachment anxiety and partners’ experiences of power

Attachment avoidance should also shape their partner’s power. Indeed, the primary goal of the distancing and withdrawal emerging from attachment avoidance is to reduce partners’ influence to retain control over one’s own needs and goals (see Overall, 2019). In particular, the principal strategy of withdrawal should downgrade the partner’s power by reducing the partner’s ability to influence actors or change actors’ behavior (Eldridge & Christensen, 2002; Overall et al., 2013). Withdrawal behaviors such as being emotionally unavailable and avoiding important discussions severely limit the partner’s ability to effectively address concerns and resolve conflicts. Moreover, withdrawal communicates a lack of investment to partners (Sasaki & Overall, 2021), and seeing actors as less invested is fundamental to whether partners feel able to influence outcomes and have power in their relationship (Oriña et al., 2011; Sprecher et al., 2006). Finally, these behavioral strategies often may constrain partners’ influence. Withdrawal may often force partners of avoidant individuals to soften influence attempts and other requests (Farrell et al., 2016; Overall et al., 2013), which may help downregulate withdrawal, but limit the partner’s ability to influence avoidant actors to reach their own goals (Overall et al., 2013). Thus, withdrawal by actors high in avoidance may lead their partners to experience lower relationship power.

## Attachment Anxiety and Power Within Close Relationships

Attachment anxiety is postulated to develop when people have experienced unpredictable, inconsistent caregiving generating beliefs that partners will eventually be rejecting (Bowlby, 1973). Such uncertainty leads to constant fears of rejection and abandonment along with strong desires for closeness, which result in people high in attachment anxiety persistently seeking reassurance and closeness (Mikulincer & Shaver, 2003). Rather than avoiding dependence as avoidant individuals do, anxious individuals become hyper-dependent on their partner’s care and support and behave in ways designed to increase closeness in relationships (Overall, 2019; Overall & Cross, 2019). For example, individuals high in anxiety are eager to disclose to their partners (Tan et al., 2012), desire high levels of support from their partner (Anders & Tucker, 2000; Collins & Feeney, 2004), and want to provide their partners support to be valued and loved in return (Feeney et al., 2013; Lemay & Spongberg, 2015). Such heightened dependence also means that anxious individuals experience intense distress when encountering relationship conflict (Campbell et al., 2005; Haydon et al., 2020), leading anxious individuals to protest against threats to the relationship bond by seeking reassurance of their partner’s care and emphasizing their hurt feelings and dependence (Overall et al., 2014; Jayamaha et al., 2016).

The specific needs, goals, and behaviors associated with attachment anxiety should shape their experience of relationship power (see Table 1). Anxious individuals’ hunger for acceptance is hard to satiate and, combined with fears that the partners are not committed, amplify dependence on intimate partners (Overall et al., 2014; Lemay & Dudley, 2009). Prominent theories of power highlight why this context will produce a sense of low power: People experience lower power when they lack control over needs and goals (Keltner et al., 2003), especially fundamental needs in relationships (Thibaut & Kelley, 1978). Anxious individuals also underestimate their partner’s commitment and satisfaction (e.g., Rodriguez et al., 2019), and perceiving partners as less invested likely limits feelings of influence and power (Oriña et al., 2011; Sprecher et al., 2006). Moreover, anxious individuals focus on supporting their partner as a means to gain and entice reciprocation of their partner’s love (Feeney et al., 2013; Overall, 2019), which reflects a lack of power over prized outcomes. Thus, actors high in attachment anxiety are likely to experience low relationship power.

Attachment anxiety may also shape the partner’s relationship power. On the one hand, partners are in the powerful position to provide the emotional resources and closeness that anxious individuals crave. Moreover, because anxious individuals want to meet the needs and goals of their partners (Feeney et al., 2013), partners may feel prioritized in their relationship, which may enhance their sense of power. On the other hand, partners are often motivated to attenuate anxious individuals’ insecure thoughts and distress by exaggerating affection and providing reassurance (e.g., Lemay & Dudley, 2011; Lemay & Ryan, 2018). Further, anxious individuals’ exaggeration of their hurt feelings and need for care induces guilt in committed partners (Jayamaha et al., 2016; Overall et al., 2014), motivating amend-making and reassurance efforts (Baumeister et al., 1994) that likely undercut the partners’ sense of power. Taken together, these potential opposing processes might produce mixed or null associations between attachment anxiety and partners’ experiences of power.

## Current Research

The current studies test the connections between two fundamental components of relationships: attachment and power. As summarized in Table 1, attachment avoidance and anxiety are characterized by core beliefs about the ability to depend on and influence partners to obtain one’s needs in relationships generating strategies to regulate actors’ own and their partner’s dependence and power. With regard to attachment avoidance, needs for control and sensitivity to partners’ dependence and influence should lead actors high in avoidance to experience lower relationship power as well as motivate defensive behavioral strategies such as withdrawal that should reduce their partners’ experience of power. With regard to attachment anxiety, intense needs and striving for closeness and reassurance should lead actors high in anxiety to experience lower relationship power. However, because partners are in a position of power to fulfill anxious individuals’ dependence-based needs but are also subjected to guilt-induction strategies that elicit reassurance, there may be mixed or null associations between attachment anxiety and partners’ power.

Despite the nature and importance of these theoretical links, prior research has not provided direct tests of these actor and partner associations. One prior study (Oka et al., 2016) combined measures of attachment anxiety and avoidance to show evidence that “insecure” attachment was associated with lower relative power. However, the associations did not specify specific links with attachment anxiety or avoidance; measures of relative power cannot distinguish lower actor power from higher perceived partner power; and the method could not test whether anxiety and avoidance were related to the partner’s experience of power. Another paper (Overall et al., 2016) reported negative correlations between attachment anxiety and avoidance and actor’s relationship power as part of supplementary analyses, but did not report correlations with partner’s power, or model actor and partner effects of attachment insecurity. Yet, another paper (Overall & Sibley, 2009) reported null associations between anxiety and avoidance and daily reports of influence and control. The current studies overcome these limitations by using established, widely-used scales to assess *both* actors’ and partners’ attachment insecurity and relationship power to provide the first systematic test of the predicted associations in Table 1 (Studies 1–3). Our studies also examine, for the first time, a behavioral process that links actors’ characteristics and behavior to partners’ power. After establishing a reliable partner effect between attachment avoidance and power, we test whether withdrawal helps explain the link between actor’s avoidance and partner’s power (Studies 4 and 5).

## Transparency and Openness

We present the studies in the order in which the data were analyzed. Studies 1 and 2 provided initial examinations that were not preregistered. Study 3 preregistered a replication of these results (https://aspredicted.org/7C9_395). Study 4 was a preregistered test of the role of withdrawal in the links between attachment avoidance and partner’s power (https://aspredicted.org/PW1_ZVD), and Study 5 replicated this test in a different context. Data and syntax are available online (https://osf.io/q82zg/). We report all measures and exclusions in these studies.

## Study 1

Study 1 involved an initial test of the predictions in Table 1 using an Israeli sample that included well-established measures of attachment anxiety and avoidance and relationship power.^1^ To show that the associations were specific to power, we also assessed and controlled for both actor’s and partner’s commitment given that commitment is a principle foundation of relationship power (Thibaut & Kelley, 1978) and associated with attachment anxiety and avoidance (Simpson et al., 1996; Tan et al., 2012).

### Method

#### Participants and Procedure

Participants were recruited via word-of-mouth advertising in the greater Tel Aviv region. Exclusion criteria were: younger than 18 years, and/or less than 1 month in the present relationship. The final sample comprised 163 woman–man couples (see Table 2 for demographics). Given that the study was already collected prior to the current analyses, we assessed *achieved power* for typical small-to-medium effect sizes with this sample size given the observed correlations of variables across partners (α = .05; correlation between errors = .12; correlations between actor and partner variables = .185, Ackerman et al., 2020). We had high statistical power (.96) to detect small–medium actor effects of β = .20 and adequate power (.79) to detect small partner effects of β = .15. The effect sizes in the present study were higher than these starting values. Participants completed an online survey independently of their partner, which included assessments of power, attachment insecurity, commitment, and other variables not germane to the current study. No prior articles have used this sample to examine actor or partner effects of attachment on power.

**Table 2.** Sample Characteristics Across Studies.

Characteristic	Study 1	Study 2	Study 3	Study 4	Study 5
Location	Israel	Germany	New Zealand	New Zealand	New Zealand
* N* Couples	163	287	517	138	151
Age					
Women (*M*, *SD*, range)	32.11 (10.00) (19 to 63)	27.23 (10.80) (18 to 60)	26.94 (9.38) (16 to 74)	24.28 (7.18) (18 to 74)	22.54 (5.88) (17 to 48)
Men (*M*, *SD*, range)	34.66 (10.38) (20 to 65)	29.08 (11.44) (18 to 67)	28.39 (10.37) (17 to 78)	25.33 (7.84) (18 to 78)	23.47 (5.93) (17 to 48)
Relationship length					
Years (*M*, *SD*, range)	8.87 (9.00) (1 month to 45 years)	4.96 (7.16) (1 month to 36 years)	5.64 (6.45) (6 months to 52 years)	3.63 (5.11) (1 year to 52 years)	2.87 (2.85) (5 months to 21 years)
Relationship status					
Married	55.2%	11.4%	34.1%	22.5%	11.9%
Engaged	5.5%	4.1%	—	—	—
Cohabiting	—	—	29.7%	29.0%	35.1%
Serious relationship	39.3%	84.5%	31.5%	40.2%	52.3%

*Note*: “—” information not gathered in that study. Couples were classified to be in a “serious relationship” if they were in established ongoing relationships of a minimum duration (>1 month in Studies 1 and 2, >6 months in Study 3, >12 months in Study 4, and >5 months in Study 5). See Supplemental Material for analyses examining whether relationship length and status moderated the actor and partner links between attachment and power.

#### Measures

All items were rated on a scale ranging from 1 (*strongly disagree*) to 7 (*strongly agree*) and were averaged to construct scores. See Table 3 for descriptive statistics and reliability.

**Table 3.** Studies 1 to 3: Descriptive Statistics, Cronbach’s Alphas, Tests of Gender Differences, and Zero-Order Correlations Across Measures.

Variable	Women			Men			*t*	*|d|*	Correlations		
	*M*	*SD*	α	*M*	*SD*	α			Avoidance	Anxiety	Power
Study 1											
Avoidance	1.94	0.93	.73	1.87	0.82	.70	−0.90	0.07	.24**	.22**	−.32***
Anxiety	3.25	1.16	.70	3.04	1.11	.57	−1.85	0.15	.26***	.13	−.19*
Power	5.85	0.75	.74	5.57	0.85	.76	−3.40***	0.27	−.34***	−.34***	.12
Study 2											
Avoidance	1.56	0.66	.72	1.69	0.71	.74	2.65**	0.16	.34***	.26***	−.50***
Anxiety	2.62	1.17	.78	2.37	1.06	.74	−2.87**	0.17	.28***	.15**	−.39***
Power	5.93	0.71	.74	5.74	0.79	.74	−3.43***	0.20	−.38***	−.37***	.24***
Study 3											
Avoidance	2.92	1.06	.78	2.77	1.01	.79	−2.43*	0.11	.13**	.19***	−.13**
Anxiety	3.07	1.12	.81	2.87	1.04	.79	−3.19**	0.14	.24***	.11*	−.28***
Power	5.34	0.98	.84	5.00	0.95	.82	−6.59***	0.29	−.23***	−.29***	.25***

*Note. t* = Results of paired samples *t* tests. Correlations within actors (e.g., actors’ avoidance and actors’ experienced power) are presented separately for women (above the diagonal) and men (below the diagonal). Correlations across partners (e.g., women’s and men’s avoidance) are presented on the diagonal.

* *p* < .05. **p < .01. ***p < .001 (two-tailed).

##### Attachment Insecurity

The *Experience in Close Relationships Scale* (Wei et al., 2007) assessed attachment insecurity. Six items assessed attachment avoidance (e.g. “I try to avoid getting too close to my partner”) and six items assessed attachment anxiety (e.g., “I need a lot of reassurance that I am loved by my partner”).

##### Power

The *Personal Sense of Power Scale* (Anderson et al., 2012) assessed power within their relationship (“In my relationship with my partner. . .”). Eight items assessed participants’ capability to influence their partner (e.g., “I can get him/her to listen to what I say;” “Even when I try, I am not able to get my way,” reverse scored; “My wishes do not carry much weight,” reverse scored).

##### Commitment

The five-item engagement scale of the *Relationship Quality Questionnaire* (Siffert & Bodenmann, 2010) assessed commitment and investment in the relationship (e.g., “I am willing to work for our partnership”).

### Analytic Strategy

We computed actor–partner interdependence models (APIMs; Kenny et al., 2006) for distinguishable (woman–man) dyads estimated with structural equation modeling (Maximum Likelihood [ML] estimation) in M*plus* 8. We accounted for shared variance between the two attachment dimensions by modeling women’s and men’s attachment avoidance and attachment anxiety as simultaneous predictors of women’s and men’s relationship power. The APIM assesses actor effects (e.g., link between women’s attachment avoidance or anxiety and women’s power) and partner effects (e.g., link between women’s avoidance or anxiety and men’s power) accounting for the interdependence in measures across women and men. When controlling for commitment, we simultaneously modeled women’s and men’s avoidance, anxiety, and commitment as predictors of actors’ and partners’ relationship power. We computed bootstrapped 95% confidence intervals (CIs; *k* = 5,000 samples) and effect sizes Δ indexing the change in power in *SD*s when attachment avoidance or anxiety changed by 1 point (see Körner & Schütz, 2021) separately for women and men (Δ_W/M_ = *b*/*SD*_W/M_). We tested for gender differences because the meaning and consequences of power may often differ across women and men (Connell, 2013; Ridgeway, 2011). For each model, we compared the fit of a saturated model (all effects freely estimated) against a model in which actor and partner effects were constrained to be equal across women and men. A nonsignificant (*p* < .20) likelihood ratio test indicates the absence of gender differences (Kenny & Ledermann, 2010; see Supplemental Material).

### Results

Table 3 presents descriptive statistics, correlations, and tests of gender differences across attachment and power measures. The results from the APIMs are shown in Table 4. Attachment avoidance was negatively associated with both actor’s and partner’s power supporting our predictions that the beliefs and behavioral strategies associated with attachment avoidance constrain actors’ experienced power and also limit the partner’s experienced power. By contrast, attachment anxiety was associated with actors’ lower power but higher partner’s power. Finally, the associations remained very similar when controlling for both actor’s and partner’s commitment (see Supplemental Material).

**Table 4.** Studies 1 to 3: Results of APIM Analyses Estimating the Actor and Partner Associations Between Attachment Insecurity and Power.

Predictor	Actor power				Partner power			
	*b*	95% CI	*p*	|Δ|	*b*	95% CI	*p*	|Δ|
Study 1								
Avoidance	−**0.24**	[−0.34, −0.13]	<.001	.32^W^ .28^M^	−**0.23**	[−0.35, −0.11]	<.001	.31^W^ .27^M^
Anxiety	−**0.12**	[−0.20, −0.03]	.007	.16^W^ .14^M^	**0.10**	[0.01, 0.17]	.017	.13^W^ .12^M^
Study 2								
Avoidance	−**0.39**	[−0.51, −0.29]	<.001	.54^W^ .51^M^	−**0.09**	[−0.18, −0.003]	.042	.13^W^ .12^M^
Anxiety	−**0.18**	[−0.24, −0.13]	<.001	.25^W^ .23^M^	0.04	[−0.01, 0.09]	.129	.06^W^ .05^M^
Study 3								
Avoidance	−**0.12**	[−0.18, −0.06]	<.001	.12^W^ .13^M^	−**0.08**	[−0.14, −0.02]	.006	.08^W^ .08^M^
Anxiety	−**0.22**	[−0.28, −0.16]	<.001	.22^W^ .23^M^	0.02	[−0.03, 0.08]	.404	.02^W^ .02^M^

*Note. b* = Unstandardized regression coefficient, CI = Bootstrapped 95% confidence interval, Δ = Effect size, W = women, M = men. Effects were pooled across women and men unless a model with different effects for women and men was preferred according to Likelihood-Ratio tests. The bold values indicate significant *b* coefficients.

## Study 2

The results of Study 1 were consistent with predictions outlined in Table 1. In Study 2, we aimed to replicate these associations in a different cultural context (i.e., Germany). Commitment was not measured in this study and so not available for control analyses.^2^

### Method

#### Participants and Procedure

Participants were recruited via the snowball principle in Southern and Eastern Germany. Exclusion criteria were: younger than 18 years, and/or less than 1 month in the present relationship. The final sample comprised 287 woman–man couples (see Table 2). Assessing achieved power for typical small–medium effect sizes with this sample size when variables were correlated across partners as they were in the present study (α = .05; correlation between errors = .24; correlations between actor and partner variables = .245, Ackerman et al., 2020) revealed that we had high statistical power (.999) to detect small–medium actor effects (β = .20) and high power (.96) to detect small partner effects (β = .15). The effect sizes in the present study were higher than these starting values. Independently of their partner, participants completed an online survey that included assessments of power and attachment. No prior articles have used this sample to examine actor or partner effects of attachment on power.

#### Measures

##### Attachment Insecurity and Power

Participants completed the same scales as in Study 1 to assess attachment insecurity and power.

### Results

Table 3 presents descriptive statistics, reliabilities, and tests of gender differences. We applied the same analytic strategy as Study 1. The results of the APIM analyses are shown in Table 4. Replicating Study 1, attachment avoidance was negatively associated with both actor’s and partner’s power, supporting that avoidant individuals perceive themselves as low in power and may also respond in ways that downregulate their partner’s power. Attachment anxiety was negatively associated with actor’s power but unrelated to partner’s power supporting that heightened concerns for closeness and reassurance undermine anxious individual’s own (but not their partner’s) relationship power.

## Study 3

The goal of Study 3 was to replicate the results of Studies 1 and 2 in another cultural context, including illustrating that the attachment–power links were independent of commitment. Hypotheses, measures, sample, and analysis strategy were preregistered (https://aspredicted.org/7C9_395).

### Method

#### Participants and Procedure

To maximize power, we integrated data from five dyadic studies collected in New Zealand (see Sasaki et al., 2022). As preregistered, to replicate the APIMs for distinguishable dyads testing for gender differences in Studies 1 and 2, we did not include six woman–woman couples (and the small number of woman–woman couples did not allow for multi-group analysis; see further consideration of this issue in the Discussion section). The final sample comprised 517 woman–man couples (see Table 2 for sociodemographics). Assessing achieved power for typical small–medium effect sizes with this sample size when variables were correlated as they were in this study (α = .05; correlation between errors = .25; correlations between actor and partner variables = .12, Ackerman et al., 2020) revealed virtually perfect power to detect small–medium actor effects (β = .20) and high power (.999) to detect small partner effects (β = .15). In each study, couples completed the same measures of attachment and power as part of a larger battery of questionnaires. No prior articles have used these data to examine the links between attachment and power.

#### Measures

See Table 3 for descriptives, correlations, and reliabilities.

##### Attachment Insecurity

The *Adult Attachment Questionnaire* (Simpson et al., 1996) assessed attachment insecurity with reference to romantic relationships in general. Eight items assessed attachment avoidance (e.g., “I’m not very comfortable having to depend on romantic partners”) and nine items assessed attachment anxiety (e.g., “I often worry that my romantic partners don’t really love me”; 1 = *strongly disagree*, 7 = *strongly agree*).

##### Power

Participants completed the same measure used in Studies 1 and 2.

##### Commitment

Participants rated seven items from the *Investment Model Scale* (Rusbult et al., 1998) assessing their commitment (e.g., “I am committed to maintaining our relationship; 1 = *strongly disagree*, 7 = *strongly agree*).

### Results

We applied the same APIM approach as in Studies 1 and 2 (see Table 4). Attachment avoidance was negatively associated with both actor’s and partner’s power, whereas attachment anxiety was negatively associated with actor’s power but unrelated to partner’s power.^3^ The links between attachment and power remained very similar when controlling for both actor’s and partner’s commitment (see Supplemental Material). Thus, the expected links emerged in a different cultural context using a measure that assessed attachment insecurity within romantic relationship in general rather than within the specific relationship as in Studies 1 and 2.

## Study 4

Studies 1 to 3 provided new evidence that attachment insecurity is not only linked to actors’ own experience of power, but that attachment avoidance is associated with partners’ lower power. This novel partner effect demonstrates the dyadic nature of power: Power involves the relational dynamics between two people and thus core relational beliefs and associated behavioral strategies not only relate to actors’ experience of power but appear to constrain the power of their partner. In Study 4, we expand upon this novel demonstration of the dyadic nature of attachment and power by assessing the core behavioral process that we theorized would link attachment avoidance and partner power—avoidant actors’ withdrawal behavior (see Table 1). We focused on withdrawal because withdrawal (1) is the principal behavior enacted by avoidant individuals, particularly in attachment-relevant situations when power is challenged (e.g., Fraley & Shaver, 1998; Simpson et al., 1992, 1996; Overall & Sibley, 2009; Overall et al., 2013), (2) is theorized to involve managing power and constraining the partner’s influence within the communication literature (see Eldridge & Christensen, 2002), and thus (3) prior theoretical work has proposed that actors high in attachment avoidance exhibit higher withdrawal as a means to sustain control and reduce their partner’s power (Overall & Cross, 2019; Overall, 2019). In short, actors high in attachment avoidance exhibit higher withdrawal as one key way to sustain or restore control, which should limit their partner’s influence and sense of power (see Table 1).

We test this power regulation process by examining whether actors’ attachment avoidance is associated with greater withdrawal during power-relevant relationship interactions (i.e., conflict), and greater withdrawal is in turn linked to partner’s lower power. We tested this correlational pathway in two ways. First, we assessed the degree to which partners perceived actors typically engaged in withdrawal during conflict interactions along with their general sense of power as assessed in Studies 1 to 3 (Test 1). Second, we tested the hypothesized power regulation process within couples’ actual power-relevant interactions by gathering observational assessments of withdrawal and partners’ experience of conflict-related power within couples’ conflict discussions in the laboratory (Test 2). Hypotheses, measures, sample, and analysis strategy were preregistered (https://aspredicted.org/PW1_ZVD).

### Method

#### Participants

The data were drawn from one of the studies that provided questionnaire measures included in Study 3, but also had additional measures that enabled testing of the behavioral pathway contributing to the association between actors’ avoidance and partners’ power. The associations reported here do not repeat those reported in Study 3 (and have not been reported previously). Test 1 uses the same measures of attachment avoidance and sense of power, but specifically examines the mediating role of withdrawal. Test 2 has no overlap by examining both withdrawal and power within couples’ conflict interactions. The final sample comprised 138 woman–man couples (see Table 2 for sociodemographics). Assessing achieved power when variables were correlated across partners as they were in the current study (α = .05; correlation between errors = .25; correlations between actor and partner variables = .12, Ackerman et al., 2020) revealed that we had high power (.93) to detect small–medium actor effects (β = .20) but somewhat low power (.73) to detect small partner effects (β = .15). However, achieved power for the partner effect was acceptable (.81) when using a starting value of the average effect size from Studies 1 to 3 (average β for actor avoidance to partner power = .165).

#### Procedure

During a lab-based session, couple members independently completed questionnaire-based measures of attachment, withdrawal during conflicts, and relationship power. Then, they identified three serious issues that had caused conflict in their relationship. After a warm-up discussion about non-conflictual events from the past week, couples engaged in a 7-min video-recorded discussion focused on their most serious ongoing conflict. Immediately after the discussion, each partner rated their experience of power during the interaction. Later, trained coders independently rated the degree to which each partner exhibited withdrawal during the discussion. Couples received NZ$100 for their participation.

#### Measures

See Table 5 for descriptives and reliabilities of all measures.

**Table 5.** Study 4: Descriptive Statistics, Cronbach’s Alphas, Tests of Gender Differences, and Zero-Order Correlations Across Measures.

Variable	Women			Men			*t*	*|d|*	Correlations				
	*M*	*SD*	α	*M*	*SD*	α			1.	2.	3.	4.	5.
1. Avoidance	2.87	0.98	.77	2.87	0.98	.77	−0.02	0.00	.11	−.10	−.17*	−.09	−.03
Test 1: general reports of withdrawal and power in relationships													
2. Perceived partner withdrawal	3.23	1.78	.88	3.11	1.66	.81	−0.56	0.05	.17*	.01	−.06	−.05	−.07
3. Power	5.36	0.89	.82	5.08	1.02	.86	−2.73**	0.23	−.16	−.33***	.19*	.01	.18*
Test 2: withdrawal and power during conflict interactions													
4. Observed withdrawal	1.18	0.45	—	1.37	0.80	—	2.41*	0.21	−.13	−.15	−.09	.81***	−.04
5. Conflict-related power	4.80	1.45	.79	4.97	1.38	.83	1.08	0.09	−.02	−.08	.10	.02	.17

*Note. t* = Results of paired samples *t* tests. Correlations within actors (e.g., actors’ avoidance and actors’ experienced power) are presented separately for women (above the diagonal) and men (below the diagonal). Correlations across partners (e.g., women’s and men’s avoidance) are presented in the diagonal. Test 1 involved general self-report measures of perceived partner withdrawal and power in relationships. Test 2 involved independent observers rating the degree to which each partner exhibited withdrawal during couples’ conflict discussions, and how much conflict-related power each partner felt they had at the end of their discussion.

* *p* < .05. **p < .01. ***p < .001 (two-tailed).

##### Attachment Insecurity

The *Adult Attachment Questionnaire* (Simpson et al., 1996) assessed attachment avoidance and anxiety. As preregistered, we focused specifically on attachment avoidance.^4^

### Test 1: General Reports of Withdrawal and Power

#### Perceived Partner Withdrawal

Participants completed two items assessing how their partner behaved when discussing areas of conflict: “When discussing difficulties or areas of conflict, MY PARTNER. . . (a) “withdraws or disengages from me” and (b) “is emotionally distant from me” (1 = *not at all*, 7 = *very much*).^5^

#### Power

Participants completed the same scale as in Studies 1 to 3 to assess relationship power.

### Test 2: Withdrawal and Power During Conflict Interactions

#### Observed Withdrawal Behavior

Three trained coders unaware of the current aims independently rated how much each person (a) withdrew or disengaged from their partner and (b) was emotionally distant from their partner (1 = *not at all*, 7 = *very much*). Coders showed high agreement (ICCs > .86). Ratings for each indicator of withdrawal were averaged across coders, and then the two items averaged (*r* = .73, *p* < .001).

#### Conflict-related Power

At the end of the conflict discussion, each couple member rated four items assessing the degree to which they had power to manage or influence the outcomes of the conflict discussion: “There was little I could do to solve this issue,” “I was able to do the things needed to settle this issue,” “I had little control over this issue,” and “I had the capability to solve this issue” (1 = *strongly disagree*, 7 = *strongly agree*).

#### Data Analysis Strategy

**Figure 1.**
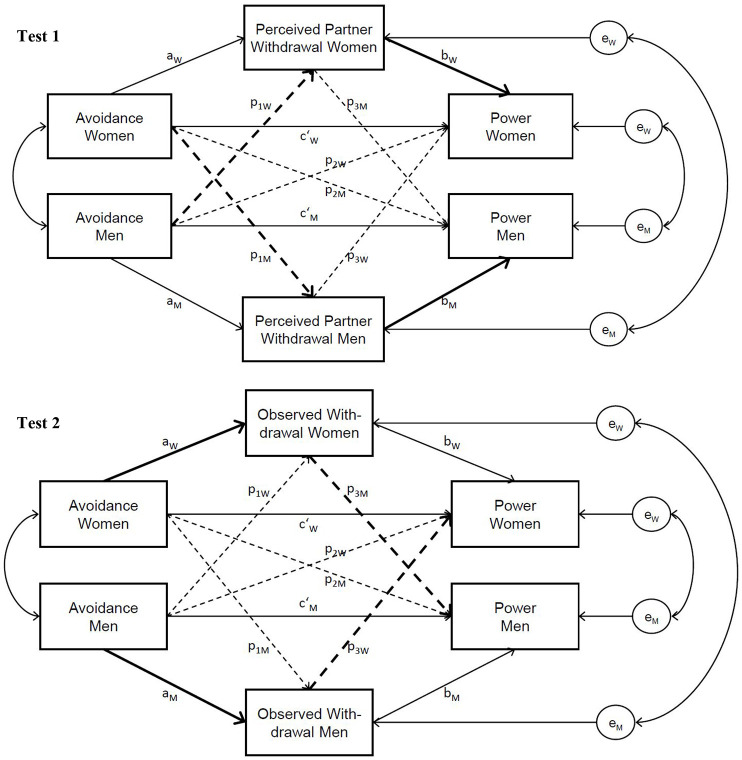
Model specification for the APIMeMs estimating the paths between attachment avoidance, withdrawal, and partner’s power (study 4). *Note.* Solid paths = actor effects. Dashed paths = partner effects. Bold paths = hypothesized mediation. In Study 4, actors’ withdrawal was represented by partners’ perceptions of actors’ withdrawal in Test 1 and observer ratings of withdrawal in Test 2.

We computed two actor–partner interdependence mediator models (APIMeMs; Ledermann et al., 2011) estimated with structural equation modeling (ML estimation) in M*plus* 8 using general reports of partners’ perceptions of actors’ withdrawal and partner’s power in relationships (Test 1) and observer ratings of withdrawal and partner’s reports of conflict-related power (Test 2). As shown in Figure 1, all actor effects (solid paths) and partner effects (dashed paths) were modeled to test the predicted links shown in bold. For Test 1, we expected higher actor attachment avoidance to predict partners’ higher perceptions of actors’ withdrawal (path *p*1 in bold), and partners’ higher perceptions of actors’ withdrawal to predict lower partners’ power (path *b* in bold). For Test 2, we expected higher actor avoidance to predict greater observer-rated actors’ withdrawal (path *a* in bold), and actors’ withdrawal to predict lower partners’ power (path *p* in bold). We computed bootstrapped 95% CIs (*k* = 5,000 samples) and calculated effect sizes Δ (=*b*/*SD*_W/M_). As in the previous studies, we compared the fit of a saturated model (all effects freely estimated) against a model in which actor and partner effects were constrained to be equal across women and men to assess gender differences (Kenny & Ledermann, 2010).

### Results

#### Test 1

The results of the APIMeM testing the associations between actors’ attachment avoidance, partner’s perceptions of actor’s withdrawal, and partners’ relationship power are shown in Table 6. The effects differed across women and men, with the expected links emerging for men. Men’s avoidance was positively linked to men’s withdrawal as perceived by women (see Partner *p*1 in bold), which in turn was negatively linked to women’s relationship power (Actor *b*): the indirect effect linking men’s avoidance, women’s perceptions of men’s withdrawal, and women’s power was negative and significant (see bottom Table 6). By contrast, women’s attachment avoidance was associated with men experiencing lower power (see Partner *p*2), but there was no evidence that withdrawal played a mediating role in this partner effect.

**Table 6.** Study 4, Test 1: Results of APIMeM Analyses Estimating the Actor and Partner Associations Between Attachment Avoidance, Perceived Partner Withdrawal, and Power.

Direct and indirect effects	*b*	95% CI	*p*	Δ
Direct effects				
Avoidance → perceived partner withdrawal				
Actor (*a*)	−0.03^W^ **0.29**^M^	[−0.34, 0.29]^W^ [0.02, 0.57]^M^	.867^W^ .046^M^	−.02^W^ .17^M^
Partner (*p*_1_)	0.03^W^ **0.52**^M^	[−0.27, 0.29]^W^ [0.24, 0.81]^M^	.841^W^ <.001^M^	.02^W^ .31^M^
Perceived partner withdrawal → power				
Actor (*b*)	−**0.09**^W^ −**0.20**^M^	[−0.17, −0.02]^W^ [−0.31, −0.08]^M^	.016^W^ .001^M^	−.10^W^ −.20^M^
Partner (*p*_3_)	−0.06^W^ 0.02^M^	[−0.16, 0.04]^W^ [−0.06, 0.11]^M^	.247^W^ .566^M^	−.07^W^ .02^M^
Avoidance → power				
Actor (*c*’)	−0.14^W^ −0.06^M^	[−0.29, 0.01]^W^ [−0.23, 0.11]^M^	.065^W^ .519^M^	−.16^W^ −.06^M^
Partner (*p*_2_)	−**0.20**^W^ −0.08^M^	[−0.38, −0.02]^W^ [−0.26, 0.10]^M^	.028^W^ .406^M^	−.22^W^ −.08^M^
Indirect partner effects				
Total	−**0.20**^W^ −0.12^M^	[−0.388, −0.017]^W^ [−0.306, 0.065]^M^	.035^W^ .211^M^	
Total indirect	0.00^W^ −0.04^M^	[−0.061, 0.067]^W^ [−0.108, 0.011]^M^	.901^W^ .161^M^	
Specific indirect	−0.01^W^ −**0.05**^M^	[−0.060, 0.055]^W^ [−0.110, −0.011]^M^	.848^W^ .048^M^	

*Note.* Notation for direct effects refer to paths in Figure 1: Test 1, top panel. *b* = Unstandardized regression coefficient, CI = Bootstrapped 95% confidence interval, Δ = Effect size, W = women, M = men. Effects were pooled across women and men unless a model with different effects for women and men was preferred according to Likelihood-Ratio tests (indicated by the use of superscripts in columns *b* to *p*). The bold values indicate significant *b* coefficients or significant indirect effects (CI does not include zero).

#### Test 2

The results of the APIMeM testing the associations between attachment avoidance, observer ratings of withdrawal during couple’s conflict discussions, and conflict-related power are shown in Table 7. No gender differences emerged. As expected, greater actor attachment avoidance was linked with greater observed actor withdrawal (Actor *a*), which in turn was linked to partner’s lower power (Partner *p*3). The 95% CI as the critical test for mediation suggests that withdrawal was a correlational mediator between actor avoidance and partner power (see bottom of Table 7).

**Table 7.** Study 4, Test 2: Results of APIMeM Analyses Estimating the Actor and Partner Associations Between Attachment Avoidance, Observer Rated Withdrawal, and Power.

Direct and indirect effects	*b*	95% CI	*p*	Δ
Direct effects				
Avoidance → observed rated withdrawal				
Actor (*a*)	**0.08**	[0.01, 0.16]	.046	.18^W^ .10^M^
Partner (*p*_1_)	0.02	[−0.05, 0.09]	.605	.04^W^ .02^M^
Observer rated withdrawal → power				
Actor (*b*)	−0.08	[−0.38, 0.16]	.584	−.06^W^ −.06^M^
Partner (*p*_3_)	−**0.25**	[−0.46, −0.03]	.021	−.17^W^ −.18^M^
Avoidance → power				
Actor (*c*’)	−0.04	[−0.21, 0.13]	.613	−.03^W^ −.03^M^
Partner (*p*_2_)	−0.05	[−0.22, 0.12]	.564	−.03^W^ −.04^M^
Indirect partner effects				
Total	−0.07	[−0.239, 0.099]	.417	
Total indirect	−0.02	[−0.069, 0.001]	.196	
Specific indirect	−**0.02**	[−0.061, −0.001]	.172	

*Note.* Notation for direct effects refer to paths in Figure 1, Test 2, bottom panel. *b* = Unstandardized regression coefficient, CI = Bootstrapped 95% confidence interval, Δ = Effect size, W = women, M = men. Effects were pooled across women and men unless a model with different effects for women and men was preferred according to likelihood-ratio tests. The bold values indicate significant *b* coefficients or significant indirect effects (CI does not include zero).

## Study 5

Study 4 provided evidence that actors’ attachment avoidance was associated with actors’ greater withdrawal, as perceived by partners and observational coders, which in turn was associated with partners experiencing lower power. The aim of Study 5 was to replicate the links between attachment avoidance, perceived partner withdrawal, and partner power (Study 4, Test 1) during the ecological context of daily life. To maximize statistical power, we integrated two daily sampling studies in which each couple member completed measures assessing attachment insecurity and then reported on their partner’s withdrawal and their experience of power every day for 3 weeks.

### Method

#### Participants

Data were drawn from two studies that used identical procedures and have been previously integrated to assess the relational consequences of withdrawal (Sasaki & Overall, 2021). No prior articles have used these data to examine the links between attachment, withdrawal, and power. The sample comprised 151 couples (see Table 2 for demographics). Evaluating achieved power with this sample size when variables were correlated across partners as they were in this study (α = .05; correlation between errors = .415; correlations between actor and partner variables = .09, Ackerman et al., 2020) indicated that we had high power (.95) to detect small–medium actor effects (β = .20) and adequate power (.78) to detect small partner effects (β = .15). The sample met recommendations of at least 100 clusters to detect small between-level effects (Meulemann & Billiet, 2009).

#### Procedure and Measures

At an in-person research session, participants independently completed baseline questionnaires, including demographics and attachment insecurity, and were given detailed instructions for completing a web-based daily record every day for the following 21 days. Participants completed on average 19 daily entries (total entries = 5,771).

##### Attachment Insecurity

Participants completed the same scale as in Studies 3 and 4.

##### Daily Partner Withdrawal

Participants rated two items assessing the degree to which their partner withdrew during their interactions that day: “My partner withdrew from me and did their own thing,” and “My partner seemed like they wanted to be left alone and/or spend less time with me” (1 = *not at all* to 7 = *extremely*). Both items were highly correlated for women and men when aggregated across days (*r*s = .84/.83, *p*s < .001).^6^

##### Daily Power

Power was assessed with a single item that differed across the two integrated samples. In one sample, participants rated “I did NOT have power or control when interacting with my partner” (1 = *not at all* to 7 = *extremely*), which was reverse-coded so that higher scores reflect higher daily power. In the other sample, participants rated “I had a lot of power or control when interacting with my partner” (1 = *not at all* to 7 = *extremely*). The results showed the same pattern when analyzing the samples separately (see Supplemental Material), so we present the higher-powered analysis integrating the samples.

#### Analytic Strategy

We computed APIMeMs (Ledermann et al., 2011) with multilevel structural equation modeling (Preacher et al., 2010) in M*plus* 8 (Bayesian estimation with noninformative priors). Daily reports (*N* = 3,171, level 1) were nested in dyads (*N* = 151, level 2). As in Study 4 Test 1, actors’ attachment avoidance (grand-mean centered) was specified as a predictor of partners’ perceptions of actors’ daily withdrawal (mediator) and partner’s daily power (outcome). Attachment avoidance is a level 2 time-constant variable with a between-component only, whereas withdrawal and power are level 1 variables with between- and within-components (Preacher et al., 2010). At the within level, we modeled same-day effects because we expect that withdrawal should immediately limit partner’s power within specific interactions each day. These within-interaction processes should produce average patterns that account for why between-person differences in attachment avoidance produce between-person differences in partner’s perceived withdrawal and power. Thus, the mediation effect is tested at the between-level. The model follows Test 1 in Figure 1: Actors’ higher attachment avoidance is expected to be associated with partners’ higher perceptions of actors’ daily withdrawal on average (path *p*1) and in turn partners’ lower power on average (path *b*). Significance of the indirect effect was tested by computing 95% CIs (credibility intervals). Model fit was best when paths were constrained to be equal than freely estimated across women and men (see Supplemental Material). Thus, the effects presented are pooled across women and men.^7^

### Results

See Table 8 for descriptive statistics and Table 9 for the results of the multilevel structural equation model. Greater actors’ attachment avoidance was associated with greater actor’s withdrawal perceived by partners (see Partner *p*1) and partners’ greater perceived withdrawal in turn was associated with partner’s lower power (Actor *b*). Withdrawal was a significant mediator as the 95% CI of the indirect effect did not include zero (see bottom Table 9). Thus, Study 5 replicated the results of Study 4 within the context of couples’ daily lives, supporting that avoidant actors’ withdrawal (as perceived by partners) helps to explain the links between actors’ avoidance and their partner’s experience of lower power.

**Table 8.** Study 5: Descriptive Statistics, Cronbach’s Alphas, Tests of Gender Differences, and Zero-Order Correlations Across Measures Based on Aggregated Person-Level Means.

Variable	Women			Men			*t*	*|d|*	Correlations		
	*M*	*SD*	α	*M*	*SD*	α			1.	2.	3.
1. Avoidance	3.02	1.06	.76	2.79	0.90	.72	2.08*	0.17	.09	.25**	−.16
2. Perceived partner withdrawal	2.03	0.95	.94	1.91	0.81	.94	1.88	0.15	.18*	.56***	−.46***
3. Power	5.66	1.08	.95	5.50	1.06	.95	1.49	0.12	−.11	−.40***	.27***

*Note.* Reliability of the daily measures of perceived partner withdrawal and power were computed as within-person reliability (i.e., consistency across days). *t* = Results of paired samples *t* tests. Correlations within actors (e.g., actors’ avoidance and actors’ experienced power) are presented separately for women (above the diagonal) and men (below the diagonal). Correlations across partners (e.g., women’s and men’s avoidance) are presented in the diagonal.

* *p* < .05. **p < .01. ***p < .001 (two-tailed).

**Table 9.** Study 5: Results of Multilevel APIMeM Analyses Estimating the Actor and Partner Associations Between Attachment Avoidance, Perceived Partner Withdrawal, and Power.

Direct and indirect effects	Estimate	95% CI	*p*
Within level			
Perceived partner withdrawal → power			
Actor (*b*_w_)	−**0.17**	[−0.20, −0.14]	<.001
Partner (*p*_w3_)	−**0.05**	[−0.08, −0.02]	<.001
Between level			
Avoidance → perceived partner withdrawal			
Actor (*a*)	**0.17**	[0.07, 0.27]	<.001
Partner (*p*_1_)	**0.16**	[0.05, 0.24]	<.001
Perceived partner withdrawal → power			
Actor (*b*)	−**0.55**	[−0.72, −0.40]	<.001
Partner (*p*_3_)	0.02	[−0.19, 0.19]	.860
Avoidance → power			
Actor (*c*’)	−0.04	[−0.16, 0.07]	.520
Partner (*p*_2_)	0.10	[−0.04, 0.23]	.120
Indirect effect			
Avoidance → partner withdrawal → partner power (*p*_1_*b)	−**0.08**	[−0.152, −0.029]	<.001

*Note*. Notation for direct effects refer to paths in Figure 1, Test 1. CI = 95% credibility interval. Effects were pooled across women and men. The bold values indicate significant estimates or significant indirect effects (CI does not include zero).

## General Discussion

The present studies provide the first systematic analysis of the interplay between two foundational relational constructs: attachment and power. Attachment avoidance and anxiety reflect fundamental beliefs about dependence and influence in relationships and thus should shape experiences of power (see Table 1). Illustrating that core relational characteristics are linked to relationship power, Studies 1 to 3 showed that greater attachment avoidance and anxiety were associated with experiencing lower relationship power. Emphasizing the dyadic nature of both attachment and power, greater attachment avoidance was also associated with partners’ experiencing lower power. These associations replicated across different cultures and continents (Israel, Germany, New Zealand) and different attachment measures.

Illustrating that attachment avoidance is associated with partners’ relationship power underscores that more attention should be paid to dyadic processes that shape relationship power. The withdrawal behavior that avoidant actors exhibit reflect attempts to limit their partner’s influence (Overall, 2019). In Studies 4 and 5, we tested this dyadic power regulation process. Actors’ higher avoidance was associated with partner’s perceiving actor’s greater withdrawal and in turn partner’s experiencing lower power within couples’ conflict interactions (Study 4) and daily life (Study 5). This correlational pathway was also supported when analyzing observer reports of withdrawal during lab-based conflict interactions (Study 4). These findings provide a novel demonstration that key relationship behaviors are likely enacted to successfully regulate the power of relationship partners. Below, we clarify how the current results advance understanding of both attachment and power.

### Theoretical Advances and Implications

The present studies demonstrate that personal characteristics should be more fully integrated in existing power theories. Theories of power primarily focus on power emerging from structural differences (i.e., positions of power) that afford control over valued outcomes (Keltner et al., 2003; Magee & Smith, 2013; Guinote, 2017). The dyadic power-social influence model (Simpson et al., 2015) emphasized the importance of personal characteristics like attachment insecurity in shaping relationship power, but did not detail specific links with actor’s or partner’s power. Other theoretical treatments suggested that attachment anxiety and avoidance reflect distinct power-related concerns and behaviors (Cross et al. 2019; Overall, 2019), but no prior studies have tested these links. Guided by an integration of attachment and power theories (see Table 1), the current results demonstrate that personal characteristics that capture differences in managing dependence and control in close relationships relate to how relationship power is experienced. These findings indicate that other dispositions or attitudes related to concerns about dependence and power should shape experiences of power (e.g., Cross et al., 2019) within close and non-close relationships.

The present findings also uniquely highlight that characteristics of relationship partners are also associated with relationship power. Indeed, power is a socio-relational construct (Anderson et al., 2012) and thus should be influenced by the person who provides the social context in which power is experienced. Assessing these types of partner effects requires applying a dyadic approach that differentiates actor and partner effects. Yet, with few exceptions (e.g., Körner & Schütz, 2021, 2024; Overall et al., 2023), research on power rarely differentiates between actor and partner power and, thus, cannot identify how actors’ characteristics and power can influence partners’ or vice versa. Partner effects may be stronger in ongoing relationships, in which actors influence partners across repeated interactions, but distinguishing actor from partner power is just as important in understanding the emergence of power in non-close relationships (Overall et al., 2023) or new dyads constructed in the laboratory by assigning people to specific roles (Anderson & Galinsky, 2006; Lammers et al., 2008). Even in newly formed dyads, actors’ characteristics will alter how they experience power, but (as discussed next) they also are likely to determine behavioral responses that alter the power experienced by their social partner.

Our dyadic approach, theoretical analysis (Table 1), and current findings illustrate that common, important behaviors (i.e., withdrawal) are likely enacted to regulate other’s power. The power literature predominantly focuses on the behavioral consequences of experienced power (e.g., Magee & Smith, 2013; Guinote, 2017) rather than how behaviors enacted in relationship interactions may shape other’s power. We provided unique evidence for an interpersonal power regulation process: actors high in avoidance engaged in greater withdrawal as perceived by partners and observational coders, which in turn was associated with their partner experiencing lower power. Withdrawal is a quintessential behavioral strategy associated with attachment avoidance (Overall et al., 2013) and power (Eldridge & Christensen, 2002) and has been described as a way avoidant individuals avoid dependence and maintain control by limiting their partners’ power (Overall & Cross, 2019; Overall, 2019). The current results provide novel evidence of this power regulation function, which has important implications for power theories. Developing current theories by incorporating the power regulation function of social behaviors (such as withdrawal) will more accurately reflect the constraints actors likely often place on other’s power in dyads and groups.

Finally, these findings have important practical implications. The negative link between attachment avoidance and partner power will have important downstream consequences including reducing partner’s relationship satisfaction and stifling partners’ pursuit of their own needs and goals (Körner & Schütz, 2021; Overall et al., 2023). Moreover, avoidant actors’ success in limiting their partner’s influence may reinforce their withdrawal behavior and sustain power regulation cycles that undermine relationship quality. Thus, interventions could be enhanced by incorporating a focus on addressing power issues and the behavioral processes that undermine partner’s power (Jenks et al., 2024). Recognizing and addressing power regulation processes is also important within broader settings, such as in workplace settings where actors’ goal of downregulating others’ power conflicts with hierarchical roles.

### Strengths, Caveats, and Future Research Directions

The current studies had many strengths. The links between attachment insecurity and power replicated across five dyadic studies from three different countries (Israeli, German, New Zealand), different attachment and power measures, participant and observer reports of behaviors, and various settings (online, lab-based, daily life). The studies incorporating 1,256 couples provided replicated evidence that one person’s attachment insecurity reliably predicts the other partner’s power, and three tests in two studies provided consistent evidence that withdrawal behavior plays an important explanatory role in this partner effect.

Despite revealing across-partner associations, the cross-sectional data limit causal conclusions. Consistent with a bulk of theory and research, attachment avoidance as a relatively stable person-level variable is more likely to motivate specific behaviors (withdrawal) and associated partner experiences (power) within couples’ interactions compared to withdrawal or partner’s power producing attachment avoidance. Yet, the success of withdrawal constraining partner’s power may reinforce the power regulation processes associated with attachment avoidance. In particular, our theorizing and others (e.g., Eldridge & Christensen, 2002; Overall, 2019) suggests that withdrawal is an effective strategy to limit partners’ influence. Such success may motivate increased use of this strategy. Additionally, actors can be less responsive to low power partners because low power partners are less able to affect outcomes (Overall et al., 2023), which could manifest in withdrawal. Additional analyses in Study 5 (see Footnote 7) found no lagged across-day links beyond the same-day associations between withdrawal and partner power, likely because any causal links occur within relevant interactions. Thus, future studies using experimental methods or time-series analyses across specific interactions are needed to provide stronger causal evidence for the theorized pathway and test possible reciprocal links between withdrawal and partner’s power.

Future studies examining power regulation processes will benefit from multiple assessments of power-relevant behaviors. In the current studies, withdrawal as perceived by partners and objective observers linked attachment avoidance to partners’ power, whereas avoidant actors’ reported withdrawal did not (see Supplemental Material). We theorized and preregistered that partners’ power would be lower to the extent that they perceived avoidant actors to withdraw. Thus, avoidant actors’ withdrawal may only relate to partners’ power if partners detect withdrawal behavior. It is also possible that actors’ reports of their own behavior may be biased by self-enhancement or unawareness of how their avoidance is expressed. Providing support that partner’s perceptions of withdrawal captured actual behavioral dynamics, the links between actors’ avoidance and partners’ power via actors’ withdrawal was supported by observational coding (Study 4). Thus, a multi-informant approach provides converging evidence and insight into the psychological processes explaining partner effects on power.

Future research could also tackle explanations for the mixed associations between attachment anxiety and partner power. Although greater anxiety was associated with greater partner power in Study 1, this was not case in the other studies. These mixed, mostly null, findings may be due to opposing processes (see Table 1). Partners have the power to provide the love and affection that individuals high in anxiety crave, potentially leading partners to feel prioritized (Feeney et al., 2013) and perceiving greater influence. On the other hand, anxious individuals often engage strategies that force partners to express love and affection (Overall et al., 2014; Lemay & Dudley, 2011), likely reducing partner’s sense of power. Future investigations taking a dyadic approach could examine a multiple mediator suppression model involving attachment anxiety amplifying partner’s felt prioritization and in turn power (pathway 1) countered by strategies eliciting partner reassurance and in turn lower power (pathway 2). Notably, these potential pathways continue to highlight that aspects of actors may generate dyadic processes that shape their partner’s power.

Future studies could also expand on power regulation processes by examining other important power-related behaviors. Other distancing behaviors, such as restricted self-disclosure and support seeking, likely contribute to the effects of attachment avoidance and withdrawal examined here. Other damaging behaviors may also reflect power regulation. Aggression emerges when competitive power beliefs motivate efforts to restore or gain power (Cross et al., 2019), and expressing anger can help to restore actors’ felt power by changing, ceasing, or reducing the influence of their partner’s behavior (Overall et al., 2013). However, no prior studies have tested whether aggression shifts power across partners during dyadic interactions or across time. Many other behaviors may be enacted to shift or gain power, including seemingly pro-social behaviors (e.g., sacrifice, support, ingratiation) that are motivated to maintain connections with high power partners (e.g., Overall et al., 2023). Examining how a greater range of behaviors solidify or change actors’ and partners’ power will advance understanding of how people manage power in social relationships.

Finally, future studies should expand the sociodemographics of our samples. Our research focused on woman–man couples, revealing very few gender differences in the links between attachment and power. However, theoretical models of gender and power (Connell, 1987; Ridgeway, 2011) suggest that the meaning and operation of power may differ across relationship contexts. In woman–man couples, traditional gender roles may be more likely to shape relationship processes, whereas same-gender couples may negotiate power and conflict in more egalitarian ways (Kurdek, 2004; Peplau & Fingerhut, 2007). We recognize the importance of expanding these studies to investigate whether dyadic power processes are similar or vary across same-gender and gender-diverse couples. Additionally, although many couples across samples were cohabiting, engaged, or married (see Table 2), it is possible that the links between attachment and power may vary in long-term couples with a history of investment and negotiated roles. Additional analyses (see Supplemental Material) provided no evidence for a moderating role of relationship status and length, but the way that dyadic attachment and power processes may shift across the development of relationships is a valuable avenue for future research. Finally, the way power is experienced, responded to, and regulated may vary in collectivistic countries compared to the relatively individualistic cultures our samples were drawn (Torelli et al., 2020), which is an important future direction.

### Conclusion

Power is inherently dyadic. Core relational beliefs and associated behavioral strategies should not only influence actors’ experience of power but also constrain their partner’s power. We illustrate the importance of a dyadic approach to link relational characteristics with power regulation processes in five studies. As expected, greater attachment avoidance and anxiety were associated with lower actor power. Additionally, greater attachment avoidance was associated with partner’s experiencing lower power, and examining withdrawal during conflict and daily life provided evidence for a specific power regulation process: Avoidant actors were more likely to engage in withdrawal as perceived by partners or observed by independent coders, which in turn predicted partners experiencing lower power. These findings advance existing power and relationship theories and research by highlighting how relational characteristics and behaviors shape both actors’ and partners’ power.

## Supplemental Material

sj-docx-1-psp-10.1177_01461672251333165 – Supplemental material for The Relational Nature of Attachment and Power: Attachment Avoidance and Withdrawal Limit Partners’ Power Supplemental material, sj-docx-1-psp-10.1177_01461672251333165 for The Relational Nature of Attachment and Power: Attachment Avoidance and Withdrawal Limit Partners’ Power by Robert Körner, Nickola C. Overall, Valerie T. Chang, Matthew D. Hammond, Eri Sasaki, Astrid Schütz and Erez Zverling in Personality and Social Psychology Bulletin

## Notes

1. This study also had a measure of relative power (rating of who has more relationship power). Relative measures conflate actor and partner power and so cannot be used to test distinct associations between attachment and actors’ versus partners’ power (Körner & Schütz, 2024; Overall et al., 2023). See Supplemental Material for more information and related analyses.

2. This study also had a measure of the motive for power (i.e., desire to obtain power). See Supplemental Material for related analyses.

3. We preregistered modeling anxiety and avoidance in separate analyses as was originally conducted in Studies 1 and 2. We agreed with reviewers to replace with models simultaneously estimating effects for anxiety and avoidance. The results and conclusions were the same (see Supplemental Material).

4. Analyses examining attachment anxiety in Studies 4 and 5 are presented in the Supplemental Material. Consistent with the overall pattern in Studies 1 to 3, anxiety was not associated with partners’ power during conflict interactions or daily life.

5. The implications of withdrawal on the partner’s power should occur because partner’s experience disengagement by avoidant actors, which undercuts the influence partners can have. Accordingly, our preregistered analyses focused on partners’ perceptions of actors’ withdrawal. See Supplemental Material for analyses of actors’ reported withdrawal.

6. The links between attachment avoidance, withdrawal, and partner’s power should occur because partners experience disengagement by avoidant actors (i.e., they perceive or experience actors’ withdrawal), which undercuts the partner’s sense of power. Thus, as in Study 4, we examine partners’ perceptions of actors’ withdrawal as the relevant mediator. See Supplemental Material for analyses of actors’ reported withdrawal.

7. The Supplemental Material presents additional analyses adding across-day lagged effects between perceived partner withdrawal and power. The within-day and between-level results in Table 9 remained, and no additional lagged effects emerged consistent with our theorizing that withdrawal should have immediate same-day implications for power.
