# Supplementary material for: The Relational Nature of Attachment and Power: Attachment Avoidance and Withdrawal Limit Partners’ Power
Source: Pers Soc Psychol Bull. 2025 Apr 29;52(7):2098–115. doi: 10.1177/01461672251333165 (PMC13216567; doi:10.1177/01461672251333165)
Supplement: sj-docx-1-psp-10.1177_01461672251333165 – Supplemental material for The Relational Nature of Attachment and Power: Attachment Avoidance and Withdrawal Limit Partners’ Power [file sj-docx-1-psp-10.1177_01461672251333165.docx]

**ONLINE SUPPLEMENT**

**to**

**The Relational Nature of Attachment and Power:**

**Attachment Avoidance and Withdrawal Limit Partners’ Power**

Overview

[Scales and Items Used to Assess Attachment, Power, Withdrawal, and Control Variables (Studies 1-5) 2](#_Toc190695273)

[Deviations From Preregistration 7](#_Toc190695274)

[Results of Likelihood Ratio Tests Examining Gender as a Moderator (Studies 1-3) 8](#_Toc190695275)

[Results of APIM Models with Anxiety and Avoidance as Separate Predictors of Power (Studies 1-3) 8](#_Toc190695276)

[Control Analyses (Commitment, Avoidance, and Anxiety as Simultaneous Predictors of Power; Studies 1 and 3) 10](#_Toc190695277)

[Results Examining (a) Relationship Length and (b) Relationship Status as Moderators in the Links Between Attachment and Power (Studies 1-3) 11](#_Toc190695278)

[Examining Additional Power Assessments: Relative Power and Motive for Power 13](#_Toc190695279)

[Results Examining Relative Power (Study 1) 13](#_Toc190695280)

[Results Examining Motive for Power (Study 2) 15](#_Toc190695281)

[Results of Likelihood Ratio Tests Examining Gender as a Moderator (Study 4) 17](#_Toc190695282)

[Additional Mediation Results (Study 4: Conflict Interaction) 17](#_Toc190695283)

[Results Examining Self-Reported Withdrawal as a Mediator in the Link Between Attachment and Power (Study 4) 17](#_Toc190695284)

[Results for Attachment Anxiety (Study 4) 19](#_Toc190695285)

[Additional Mediation Results (Study 5: Dyadic Daily Diary) 22](#_Toc190695286)

[Descriptive Statistics and Reliabilities of all Variables (Study 5) 22](#_Toc190695287)

[Results of Model Fit Statistics Examining Gender as a Moderator (Study 5) 23](#_Toc190695288)

[Results Examining Avoidance as a Predictor of Perceived Withdrawal (Mediator) and Power (Outcome) For Separate Subsamples (Study 5) 23](#_Toc190695289)

[Results Examining Anxiety as a Predictor of Perceived Withdrawal (Mediator) and Power (Outcome) (Study 5) 25](#_Toc190695290)

[Results Examining Avoidance as a Predictor of Self-Reported Withdrawal (Mediator) and Power (Outcome) (Study 5) 26](#_Toc190695291)

[Results Examining Avoidance as a Predictor of Perceived Withdrawal (Mediator) and Power (Outcome) Using Dynamic Structural Equation Modelling to Analyze Lagged Effects on the Within-Level (Study 5) 27](#_Toc190695292)

[References 29](#_Toc190695293)

# Scales and Items Used to Assess Attachment, Power, Withdrawal, and Control Variables (Studies 1-5)

**Study 1**

Experienced Power

Instruction: “In the relationship with my partner….”

1. I can get him/her to listen to what I say.
2. My wished do not carry much weight.
3. I can get him/her to do what I want.
4. Even if I voice them, my views have little sway.
5. I think I have a great deal of power.
6. My ideas and opinions are often ignored.
7. Even when I try, I am not able to get my way.
8. If I want to, I get to make the decisions.

1 = disagree strongly, 2 = disagree, 3 = disagree a little, 4 = neither agree nor disagree, 5 = agree a little, 6 = agree, 7 = agree strongly

Relative Power

1. Who tells the other person what to do more often, you or this person?
2. Between you and this person, who tends to be the boss in this relationship?
3. In your relationship with this person, who tends to take charge and decide what should be done?

1 = always him/her, 4 = about the same, 7 = always me

Attachment

1. It helps to turn to my romantic partner in times of need
2. I need a lot of reassurance that I am loved by my partner
3. I want to get close to my partner, but I keep pulling back
4. I find that my partner doesn’t want to get as close as I would like
5. I turn to my partner for many things, including comfort and reassurance
6. My desire to be very close sometimes scares people away
7. I try to avoid getting too close to my partner
8. I do not often worry about being abandoned
9. I usually discuss my problems and concerns with my partner
10. I get frustrated if romantic partners are not available when I need them
11. I am nervous when partners get too close to me
12. I worry that romantic partners won’t care about me as much as I care about them

1 = not at all, 5 = very

Commitment

1. I invest in our relationship.
2. I am prepared to make a commitment to our partnership.
3. I am committed to our partnership.
4. I am not more committed to our relationship than necessary.
5. I am committed to the well-being of our partnership.

1 = disagree strongly, 5 = agree strongly

**Study 2**

Experienced Power

Instruction: “In the relationship with my partner….”

1. I can get him/her to listen to what I say.
2. My wished do not carry much weight.
3. Even if I voice them, my views have little sway.
4. I think I have a great deal of power.
5. My ideas and opinions are often ignored.
6. Even when I try, I am not able to get my way.

1 = disagree strongly, 2 = disagree, 3 = disagree a little, 4 = neither agree nor disagree, 5 = agree a little, 6 = agree, 7 = agree strongly

Power Motive

1. I have a strong urge to gain power in the relationship.
2. I enjoy having influence over my partner.
3. I would enjoy having authority over my partner.
4. I work to control my partner more than he/she controls me.
5. I try to have more influence in my relationship than my partner.
6. I like to tell my partner what to do.

1 = disagree strongly, 2 = disagree, 3 = disagree a little, 4 = neither agree nor disagree, 5 = agree a little, 6 = agree, 7 = agree strongly

Attachment

1. It helps to turn to my romantic partner in times of need
2. I need a lot of reassurance that I am loved by my partner
3. I want to get close to my partner, but I keep pulling back
4. I find that my partner doesn’t want to get as close as I would like
5. I turn to my partner for many things, including comfort and reassurance
6. My desire to be very close sometimes scares people away
7. I try to avoid getting too close to my partner
8. I do not often worry about being abandoned
9. I usually discuss my problems and concerns with my partner
10. I get frustrated if romantic partners are not available when I need them
11. I am nervous when partners get too close to me
12. I worry that romantic partners won’t care about me as much as I care about them

1 = disagree strongly, 2 = disagree, 3 = disagree a little, 4 = neither agree nor disagree, 5 = agree a little, 6 = agree, 7 = agree strongly

**Study 3**

Attachment

1. I find it relatively easy to get close to romantic partners.
2. I'm not very comfortable having to depend on romantic partners.
3. I'm comfortable having my romantic partners depend on me.
4. I don't often worry about being abandoned by my romantic partners.
5. I don't like my romantic partners getting too close to me.
6. I'm somewhat uncomfortable being too close to my romantic partners.
7. I find it difficult to trust my romantic partners completely.
8. I'm nervous whenever any of my romantic partners gets too close to me.
9. My romantic partners often want me to be more intimate than I feel comfortable being.
10. My romantic partners are often reluctant to get as close as I would like.
11. I often worry that my romantic partners don't really love me.
12. I don't often worry about my romantic partners leaving me.
13. I often want to merge completely with my romantic partners, and this desire sometimes scares them away.
14. I'm confident my romantic partners would never hurt me by suddenly ending our relationship.
15. I usually want more closeness and intimacy than my romantic partners do.
16. The thought of being left by my romantic partners rarely enters my mind.
17. I'm confident that my romantic partners love me just as much as I love them.

1 = disagree strongly, 2 = disagree, 3 = disagree a little, 4 = neither agree nor disagree, 5 = agree a little, 6 = agree, 7 = agree strongly

Experienced Power

Instruction: “In the relationship with my partner….”

1. I can get him/her to listen to what I say.
2. My wished do not carry much weight.
3. I can get him/her to do what I want.
4. Even if I voice them, my views have little sway.
5. I think I have a great deal of power.
6. My ideas and opinions are often ignored.
7. Even when I try, I am not able to get my way.
8. If I want to, I get to make the decisions.

1 = disagree strongly, 2 = disagree, 3 = disagree a little, 4 = neither agree nor disagree, 5 = agree a little, 6 = agree, 7 = agree strongly

Commitment

1. I want our relationship to last a very long time.
2. I am committed to maintaining my relationship with my partner
3. I would not feel very upset if our relationship were to end in the near future.
4. It is likely that I will date someone other than my partner within the next year
5. I feel very attached to our relationship – very strongly linked to my partner
6. I want our relationship to last forever
7. I am oriented toward the long-term future of my relationship (for example, I imagine being with my partner several years from now).

1 = disagree strongly, 2 = disagree, 3 = disagree a little, 4 = neither agree nor disagree, 5 = agree a little, 6 = agree, 7 = agree strongly

**Study 4**

Attachment

1. I find it relatively easy to get close to romantic partners.
2. I'm not very comfortable having to depend on romantic partners.
3. I'm comfortable having my romantic partners depend on me.
4. I don't often worry about being abandoned by my romantic partners.
5. I don't like my romantic partners getting too close to me.
6. I'm somewhat uncomfortable being too close to my romantic partners.
7. I find it difficult to trust my romantic partners completely.
8. I'm nervous whenever any of my romantic partners gets too close to me.
9. My romantic partners often want me to be more intimate than I feel comfortable being.
10. My romantic partners are often reluctant to get as close as I would like.
11. I often worry that my romantic partners don't really love me.
12. I don't often worry about my romantic partners leaving me.
13. I often want to merge completely with my romantic partners, and this desire sometimes scares them away.
14. I'm confident my romantic partners would never hurt me by suddenly ending our relationship.
15. I usually want more closeness and intimacy than my romantic partners do.
16. The thought of being left by my romantic partners rarely enters my mind.
17. I'm confident that my romantic partners love me just as much as I love them.

1 = disagree strongly, 2 = disagree, 3 = disagree a little, 4 = neither agree nor disagree, 5 = agree a little, 6 = agree, 7 = agree strongly

Perceived Partner Withdrawal

Instruction: When discussing difficulties or areas of conflict, I…

1. withdraw or disengage from my partner
2. am emotionally distant from my partner

1 = not at all, 7 = very much

Self-reported Withdrawal

Instruction: When discussing difficulties or areas of conflict, MY PARTNER…

1. withdraws or disengages from me
2. is emotionally distant from me

1 = not at all, 7 = very much

Observed Withdrawal (Rated by Independent Coders)

1. withdrew or disengaged from their partner
2. was emotionally distant from their partner

1 = not at all, 7 = very much

Experienced Power

Instruction: “In the relationship with my partner….”

1. I can get him/her to listen to what I say.
2. My wished do not carry much weight.
3. I can get him/her to do what I want.
4. Even if I voice them, my views have little sway.
5. I think I have a great deal of power.
6. My ideas and opinions are often ignored.
7. Even when I try, I am not able to get my way.
8. If I want to, I get to make the decisions.

1 = disagree strongly, 2 = disagree, 3 = disagree a little, 4 = neither agree nor disagree, 5 = agree a little, 6 = agree, 7 = agree strongly

Post-discussion Report of Power

1. There was little I could do to solve this issue
2. I was able to do the things needed to settle this issue
3. I had little control over this issue
4. I had the capability to solve this issue

1 = disagree strongly, 2 = disagree, 3 = disagree a little, 4 = neither agree nor disagree, 5 = agree a little, 6 = agree, 7 = agree strongly

**Study 5**

Attachment

1. I find it relatively easy to get close to romantic partners.
2. I'm not very comfortable having to depend on romantic partners.
3. I'm comfortable having my romantic partners depend on me.
4. I don't often worry about being abandoned by my romantic partners.
5. I don't like my romantic partners getting too close to me.
6. I'm somewhat uncomfortable being too close to my romantic partners.
7. I find it difficult to trust my romantic partners completely.
8. I'm nervous whenever any of my romantic partners gets too close to me.
9. My romantic partners often want me to be more intimate than I feel comfortable being.
10. My romantic partners are often reluctant to get as close as I would like.
11. I often worry that my romantic partners don't really love me.
12. I don't often worry about my romantic partners leaving me.
13. I often want to merge completely with my romantic partners, and this desire sometimes scares them away.
14. I'm confident my romantic partners would never hurt me by suddenly ending our relationship.
15. I usually want more closeness and intimacy than my romantic partners do.
16. The thought of being left by my romantic partners rarely enters my mind.
17. I'm confident that my romantic partners love me just as much as I love them.

1 = disagree strongly, 2 = disagree, 3 = disagree a little, 4 = neither agree nor disagree, 5 = agree a little, 6 = agree, 7 = agree strongly

Perceived Partner Withdrawal

1. My partner withdrew from me and did their own thing
2. My partner seemed like they wanted to be left alone and/or spend less time with me

1 = not at all, 7 = very much

Self-reported Withdrawal

1. I withdrew from my partner and did my own thing
2. I wanted to be left alone and/or spend less time with my partner

1 = not at all, 7 = very much

Experienced Power

Subsample 1: I did NOT have power or control when interacting with my partner

Subsample 2: I had a lot of power or control when interacting with my partner

1 = disagree strongly, 2 = disagree, 3 = disagree a little, 4 = neither agree nor disagree, 5 = agree a little, 6 = agree, 7 = agree strongly

# Deviations From Preregistration

**Study 3:**

We preregistered that of the 523 couples, we would analyze 521 man-women couples and remove 2 same-gender couples because we wanted to replicate the analyses for distinguishable dyads and tests of gender difference in Studies 1 and 2. However, of the 523 couples, six couples were same-gender and so the sample for analysis was 517. See method section and analytic plan in Study 3.

We also preregistered that we would model anxiety and avoidance in separate analyses as was originally conducted in Studies 1 and 2. We agreed with reviewers to replace these analyses with models simultaneously estimating effects for anxiety and avoidance. The results and conclusions were the same. See Results of APIM Models with Anxiety and Avoidance as Separate Predictors of Power (Studies 1-3) below.

Finally, we preregistered we would report 99% CIs for the model coefficients. As it is common practice to report 95% CIs in APIM models, and we have done so across the rest of the studies, we report 95% CIs. The interpretation of the results does not change according to whether we present 99% or 95% CIs (see Table 4).

**Study 4:**

We preregistered 99% CIs to evaluate the indirect effects. However, it is standard practice to report 95% CIs for mediation tests (Hayes, 2017; MacKinnon et al., 2004; Preacher & Hayes, 2008), which is especially relevant given the size of dyadic samples. Thus in the paper we report 95% CIs, and provide the 99% CIs here. The results for Test 1 using general reports of withdrawal and power support the same conclusion if 99% CIs are specified to evaluate the indirect effects (specific indirect effect for men remains significant, *b* = -0.05, 99% CI [-0.131, -0.001]; specific indirect effect for women remains non-significant, *b* = -0.01, 99% CI [-0.087, 0.082]). However, the results for Test 2 examining the conflict discussion provide weaker evidence if the indirect effects are evaluated according to 99% CIs instead of 95% CIs (specific indirect effect across gender, *b* = -0.02, 99% CI [-0.079, 0.003]).

# Results of Likelihood Ratio Tests Examining Gender as a Moderator (Studies 1-3)

**Table S1**

*Results of Likelihood Ratio Tests for APIMs Predicting Power From Attachment Avoidance and Anxiety as Simultaneous Predictors (Saturated Model vs. Equal-Actor-Equal-Partner Effects Model Presented First)*

|  | χ²(4) | *p* |
| --- | --- | --- |
| Study 1 | 3.309 | .508 |
| Study 2 | 2.725 | .426 |
| Study 3 | 3.755 | .440 |

# Results of APIM Models with Anxiety and Avoidance as Separate Predictors of Power (Studies 1-3)

The original analyses of Study 1 and 2 were conducted prior to the development of the additional studies. Those analyses modelled attachment avoidance and anxiety separately to reduce the number of parameters. The approach pre-registered in Study 3 replicated the analytic approach in those studies, and we provided models simultaneously estimating effects for anxiety and avoidance in the original supplemental materials. However, we agreed with reviewers that the models simultaneously estimating effects for anxiety and avoidance should be presented as the main analyses in the paper. Here we present the original models that estimate the effects of anxiety and avoidance separately.

In Study 1, the actor and partner effects of attachment avoidance and the actor effect of attachment anxiety remained. However, the link between attachment anxiety and partner’s higher power was not significant as it was when modelling anxiety and avoidance as simultaneous predictors. In Study 2 and Study 3, the results remained similar for both attachment avoidance and anxiety. In summary, attachment avoidance remained a significant negative predictor of actor’s and partner’s experienced power across all models. The actor effect of attachment anxiety on power also remained consistent, but the partner effect of attachment anxiety on power that was significant in Study 1 was not significant when modelling anxiety separately. These inconsistent effects are consistent with our prediction of mixed effects for attachment anxiety and partner power, and null associations between attachment anxiety and partner power across the rest of the studies.

**Table S2**

*Results of APIM Analyses Estimating the Actor and Partner Associations between Attachment and Power When Anxiety and Avoidance are Modelled as Separate Predictors of Power*

|  |  | Actor Power | | | |  | Partner Power | | | |
| --- | --- | --- | --- | --- | --- | --- | --- | --- | --- | --- |
| Predictor |  | *b* | 95% CI | *p* | Δ |  | *b* | 95% CI | *p* | Δ |
| Study 1 |  |  |  |  |  |  |  |  |  |  |
| Avoidance |  | **-0.24** | [-0.36, -0.13] | <.001 | -0.32^W^  -0.28^M^ |  | **-0.23** | [-0.35, -0.11] | <.001 | -0.31^W^  -0.27^M^ |
| Anxiety |  | **-0.13**^W^  **-0.26**^M^ | [-0.24, -0.02]^W^  [-0.38, -0.15]^M^ | .030^W^  <.001^M^ | -0.17^W^  -0.31^M^ |  | 0.02 | [-0.06, 0.10] | .592 | 0.03^W^  0.02^M^ |
|  |  |  |  |  |  |  |  |  |  |  |
| Study 2 |  |  |  |  |  |  |  |  |  |  |
| Avoidance |  | **-0.44** | [-0.56, -0.33] | <.001 | -0.62^W^  -0.56^M^ |  | **-0.14** | [-0.22, -0.05] | .003 | -0.20^W^  -0.18^M^ |
| Anxiety |  | **-0.25** | [-0.31, -0.19] | <.001 | -0.35^W^  -0.32^M^ |  | 0.01^W^  -0.07^M^ | [-0.06, 0.08]^W^  [-0.15, 0.001]^M^ | .763^W^  .068^M^ | 0.01^W^  -0.09^M^ |
|  |  |  |  |  |  |  |  |  |  |  |
| Study 3 |  |  |  |  |  |  |  |  |  |  |
| Avoidance |  | **-0.16** | [-0.21, -0.10] | <.001 | -0.16^W^  -0.17^M^ |  | **-0.12** | [-0.18, -0.06] | <.001 | -0.12^W^  -0.13^M^ |
| Anxiety |  | **-0.26** | [-0.31, -0.20] | <.001 | -0.27^W^  -0.27^M^ |  | -0.01 | [-0.07, 0.04] | .618 | -0.01^W^  -0.01^M^ |

*Note.* *b* = unstandardized regression coefficient, CI = bootstrapped 95% confidence interval, Δ = effect size. W = women, M = men. Effects were pooled across women and men unless a model with different effects for women and men was preferred according to Likelihood-Ratio tests (indicated by the use of superscripts). Study 1: *N* = 163 couples. Study 2: *N* = 287 couples. Study 3: *N* = 517 couples. The bold values indicate significant *b* coefficients.

# Control Analyses (Commitment, Avoidance, and Anxiety as Simultaneous Predictors of Power; Studies 1 and 3)

The following table presents APIM results with commitment as a control variable on the actor and partner links between attachment and power. Results are discussed in the manuscript.

**Table S3**

*Results of APIM Analyses Estimating the Actor and Partner Associations between Attachment and Relationship Power Controlling for Commitment*

|  |  | Actor Power | | | | | |  | | Partner Power | | | | |
| --- | --- | --- | --- | --- | --- | --- | --- | --- | --- | --- | --- | --- | --- | --- |
| Predictor |  | *b* | 95% CI | *SE* | *p* | \|Δ\| |  | | *b* | | 95% CI | *SE* | *p* | \|Δ\| |
| Study 1: Israel |  |  |  |  |  |  |  | |  | |  |  |  |  |
| Avoidance |  | **-0.14** | [-0.26, -0.03] | 0.06 | .017 | 0.19^W^  0.16^M^ |  | | **-0.21** | | [-0.35, -0.06] | 0.07 | .004 | 0.28^W^  0.25^M^ |
| Anxiety |  | **-0.13** | [-0.21, -0.05] | 0.04 | .002 | 0.17^W^ 0.15^M^ |  | | **0.11** | | [0.03, 0.19] | 0.04 | .007 | 0.15^W^  0.13^M^ |
| Commitment |  | **0.24** | [0.10, 0.36] | 0.07 | < .001 | 0.32^W^  0.28^M^ |  | | -0.03 | | [-0.17, 0.12] | 0.08 | .705 | 0.04^W^  0.04^M^ |
| Study 3: New Zealand | |  |  |  |  |  |  | |  | |  |  |  |  |
| Avoidance |  | **-0.08** | [-0.14, -0.01] | 0.03 | .016 | 0.08^W^  0.08^M^ |  | | **-0.06** | | [-0.12, -0.002] | 0.03 | .034 | 0.06^W^  0.06^M^ |
| Anxiety |  | **-0.21** | [-0.27, -0.16] | 0.03 | <.001 | 0.21^W^  0.22^M^ |  | | 0.04 | | [-0.02, 0.09] | 0.03 | .191 | 0.04^W^  0.04^M^ |
| Commitment |  | **0.16** | [0.05, 0.27] | 0.06 | .004 | 0.16^W^  0.17^M^ |  | | 0.07 | | [-0.03, 0.18] | 0.05 | .156 | 0.07^W^  0.07^M^ |

*Note.* *b* = unstandardized regression coefficient, CI = bootstrapped 95% confidence interval, Δ = effect size. W = women, M = men. Effects were pooled across women and men unless a model with different effects for women and men was preferred according to Likelihood-Ratio tests (indicated by the use of superscripts). Study 1: *N* = 163 couples. Study 3: *N* = 517 couples. The bold values indicate significant *b* coefficients.

# Results Examining (a) Relationship Length and (b) Relationship Status as Moderators in the Links Between Attachment and Power (Studies 1-3)

Guided by comments during the review process, we conducted analyses examining whether the links between attachment insecurity and power were moderated by relationship status and length. We focused these analyses on Studies 1-3 because these were the largest samples and thus provide the most statistical power for these tests. We repeated the APIM analyses detailed in the manuscript (Study 1, Analytic Strategy) and added interaction terms multiplying women’s and men’s avoidance and anxiety with their (1) relationship length (in months) or (2) relationship status (3-level category: “serious relationship”; “engaged” [Studies 1-2] or “cohabiting” [Study 3]”; and “married”). The results are displayed in Table 4. No significant moderation effects were found. Thus, neither relationship length nor relationship status moderated the actor and partner links between attachment insecurity and power. Note, however, that the size of the dyadic samples means we are likely underpowered to detect moderation effects (e.g., Baranger et al., 2023; Vize et al., 2023).

**Table S4**

*Studies 1-3: Results of APIMoM Analyses Estimating the Moderating Role of Relationship Length and Status on the Links Between Attachment Insecurity and Power*

|  |  | Actor Power |  |  |  | Partner Power |  |
| --- | --- | --- | --- | --- | --- | --- | --- |
| Moderation Effects | *b* | 95% CI | *p* |  | *b* | 95% CI | *p* |
| Study 1 |  |  |  |  |  |  |  |
| Avoidance * Relationship Length | -0.001 | [-0.01, 0.01] | .868 |  | 0.003 | [-0.01, 0.02] | .693 |
| Anxiety * Relationship Length | 0.001 | [-0.01, 0.01] | .693 |  | 0.001 | [-0.01, 0.01] | .839 |
| Avoidance * Relationship Status | -0.05 | [-0.16, 0.06] | .369 |  | 0.07 | [-0.06, 0.19] | .269 |
| Anxiety * Relationship Status | 0.001 | [-0.09, 0.09] | .980 |  | 0.01 | [-0.08, 0.08] | .901 |
|  |  |  |  |  |  |  |  |
| Study 2 |  |  |  |  |  |  |  |
| Avoidance * Relationship Length | 0.004 | [-0.01, 0.02] | .600 |  | 0.01 | [-0.01, 0.01] | .355 |
| Anxiety * Relationship Length | -0.002 | [-0.02, 0.01] | .711 |  | -0.002 | [-0.01, 0.01] | .570 |
| Avoidance * Relationship Status | -0.05 | [-0.20, 0.15] | .519 |  | 0.11 | [-0.01, 0.25] | .108 |
| Anxiety * Relationship Status | 0.04 | [-0.07, 0.19] | .531 |  | 0.01 | [-0.12, 0.10] | .862 |
|  |  |  |  |  |  |  |  |
| Study 3 |  |  |  |  |  |  |  |
| Avoidance * Relationship Length | 0.000 | [-0.001, 0.001] | .774 |  | 0.000 | [0.000, 0.001] | .146 |
| Anxiety * Relationship Length | -0.001 | [-0.001, 0.000] | .150 |  | 0.000 | [-0.001, 0.000] | .335 |
| Avoidance * Relationship Status | -0.04 | [-0.11, 0.04] | .356 |  | 0.04 | [-0.03, 0.11] | .327 |
| Anxiety * Relationship Status | 0.05 | [-0.01, 0.11] | .088 |  | 0.02 | [-0.04, 0.08] | .519 |

*Note.* *b* = Unstandardized regression coefficient, CI = Bootstrapped 95% confidence interval. Effects were pooled across women and men.

# Examining Additional Power Assessments: Relative Power and Motive for Power

## Results Examining Relative Power (Study 1)

Study 1 also included a measure of relative power (see section “Scales and Items Used to Assess Attachment, Power, Withdrawal, and Control Variables (Studies 1-5)”). For women, relative power (*M* = 4.53, *SD* = .98, α = .75) was associated with actors’ own greater sense of power (*r* = .12) and greater attachment avoidance (*r* = .23) but unrelated to anxiety (*r* = -.01). Similarly, for men, relative power (*M* = 3.87, *SD* = 1.00, α = .75) was associated with greater sense of power (*r* = .36), unrelated to avoidance (*r* = .04), and associated with less anxiety (*r* = -.10). Finally, relative power was negatively associated across women and men (*r* = -.43), suggesting couple members tended to agree about their relative power in the relationship.

The APIM results of attachment as a predictor of relative power are displayed in Table S5. Avoidance was positively linked to relative power for actors (*b* = 0.20, |Δ^W/M^| = 0.27^W^/0.24^M^). Thus, even though avoidance is linked to lower experienced power, avoidant individuals report that they hold more power than their partners likely because, as our results show, attachment avoidance limits their *partner’s* power.

Women’s attachment avoidance also was negatively linked to men’s relative power (*b* = -0.25, |Δ| = 0.33^W^) showing that women high in avoidance both felt they had less power than their partner as did their men partners. Again, this highlights that failing to distinguish actor and partner power make it unclear whose power (i.e., actors higher power or partners lower power) is contributing to relative power measures.

Finally, attachment anxiety was not significantly associated with relative power despite being associated with both actors and partners’ lower power. This pattern illustrates that only focusing on relative power measures misses identifying when actors and partners both feel low power, which is associated with a range of poor relationship and personal outcomes for each person (see Körner & Schütz, 2024; Overall et al., 2023).

**Table S5**

*Study 1: Results of APIM Analyses Estimating the Actor and Partner Associations Between Attachment and Relative Power*

|  |  | Actor Relative Power | | | | |  | | Partner Relative Power | | | | |  |
| --- | --- | --- | --- | --- | --- | --- | --- | --- | --- | --- | --- | --- | --- | --- |
|  |  | *b* | 95% CI | *SE* | *p* | \|Δ\| |  | *b* | | 95% CI | *SE* | *p* | \|Δ\| | |
| Avoidance |  | **0.20** | [0.04, 0.34] | 0.08 | .011 | 0.27^W^  0.24^M^ |  | **-0.25**^W^  -0.05^M^ | | [-0.42, -0.06]^W^  [-0.24, 0.14]^M^ | 0.09^W^  0.10^M^ | .007^W^  .616^M^ | 0.33^W^  0.06^M^ | |
| Anxiety |  | -0.06 | [-0.17, 0.05] | 0.06 | .307 | 0.08^W^  0.07^M^ |  | 0.08 | | [-0.04, 0.20] | 0.06 | .187 | 0.11^W^  0.09^M^ | |

*Note.* *b* = unstandardized regression coefficient, CI = bootstrapped 95% confidence interval, Δ = effect size. W = women, M = men. Effects were pooled across women and men unless a model with different effects for women and men was preferred according to Likelihood-Ratio tests (indicated by the use of superscripts). *N* = 163 couples. The bold values indicate significant *b* coefficients.

## Results Examining Motive for Power (Study 2)

Study 2 also included a measure of desire for power assessed using the power motive subscale of the *Feeling Power and Desiring Power Scales* (Murphy et al., 2022; see section “Scales and Items Used to Assess Attachment, Power, Withdrawal, and Control Variables (Studies 1-5)”). For women, a greater motive of power (*M* = 2.29, *SD* = 1.11, α = .87) was associated with a lower sense of power (*r* = -.12) as well as greater attachment avoidance (*r* = .29) and anxiety (*r* = .18). Similarly, for men, a greater motive of power (*M* = 2.30, *SD* = 1.20, α = .89) was associated with a lower sense of power (*r* = -.14) as well as greater avoidance (*r* = .27) and anxiety (*r* = .20). Motive for power also was positively associated across women and men partners (*r* = .25, *p* < .001).

The APIM results of attachment as predictor of motive for power are displayed in Table S6. Attachment avoidance was positively linked to actors’ (*b* = 0.46, |Δ| = 0.41^W^/0.38^M^) but not partners’ motive for power, which is consistent with our argument that avoidant individuals desire more influence. Attachment anxiety also was positively associated with actors’ (*b* = 0.17, |Δ| = 0.15^W^/0.14^M^) and partners’ (*b* = 0.18, |Δ| = 0.16^W^/0.15^M^) motives for power suggesting that the dynamics associated with attachment anxiety may leave both actors and partners wanting more power. Note, however, that the association between attachment avoidance and desired power was two to three times larger than the association between anxiety and desired power. Nonetheless, mediated APIM models testing whether a greater motive for power explained the links between attachment and power revealed that the actor and partner effects in the paper were independent of the motive for power (results can be found in Mplus outputs at OSF).

**Table S6**

*Study 2: Results of APIM Analyses Estimating the Actor and Partner Associations between Attachment and Power Motive*

|  |  | Actor Power Motive | | | | |  | | Partner Power Motive | | | | | |
| --- | --- | --- | --- | --- | --- | --- | --- | --- | --- | --- | --- | --- | --- | --- |
| Predictor |  | *b* | 95% CI | *SE* | *p* | \|Δ\| | |  | | *b* | 95% CI | *SE* | *p* | \|Δ\| |
| Avoidance |  | **0.46** | [0.32, 0.59] | 0.07 | <.001 | 0.41^W^  0.38^M^ | |  | | 0.04 | [-0.10, 0.17] | 0.07 | .612 | 0.04^W^  0.03^M^ |
| Anxiety |  | **0.17** | [0.08, 0.25] | 0.04 | <.001 | 0.15^W^  0.14^M^ | |  | | **0.18** | [0.09, 0.27] | 0.05 | <.001 | 0.16^W^  0.15^M^ |

*Note.* *b* = Unstandardized regression coefficient, CI = Bootstrapped 95% confidence interval, Δ = Effect size. W = women, M = men. Effects were pooled across women and men unless a model with different effects for women and men was preferred according to Likelihood-Ratio tests (indicated by the use of superscripts in columns *b* to *p*). *N* = 287 couples. The bold values indicate significant *b* coefficients.

# Results of Likelihood Ratio Tests Examining Gender as a Moderator (Study 4)

**Table S7**

*Results of Likelihood Ratio Tests for APIMeMs Predicting Power From Attachment Avoidance or Anxiety (Saturated Model vs. Equal-Actor-Equal-Partner Effects Model)*

|  | Avoidance | |  |  | Anxiety | |
| --- | --- | --- | --- | --- | --- | --- |
|  | χ²(6) | *p* |  |  | χ²(6) | *p* |
| Mediator: partner perceived withdrawal | 14.039 | .029 |  |  | 7.894 | .246 |
| Mediator: observer rated withdrawal | 5.217 | .516 |  |  | 3.876 | .693 |
| Additional analyses: self-reported withdrawal | 7.838 | .250 |  |  | 3.820 | .710 |

# Additional Mediation Results (Study 4: Conflict Interaction)

## Results Examining Self-Reported Withdrawal as a Mediator in the Link Between Attachment and Power (Study 4)

The implications of withdrawal on the partner’s power should occur because partner’s experience disengagement by avoidant actors, which undercuts the influence partners believe they have. Accordingly, our preregistered analyses focused on partners’ perceptions of actors’ withdrawal. Here we also report the analyses for actors’ self-reported withdrawal. Descriptive statistics and gender differences appear in Table S8.

Attachment avoidance was associated with greater actors’ reported withdrawal, but APIMeM analyses revealed that actor’s self-reported withdrawal behavior did not mediate the link between attachment avoidance and partners’ relationship power (see Table S9). In this model, attachment avoidance was negatively linked to both actor’s and partner’s power, replicating the results of Studies 1 to 3. Thus, as we expected and reiterated in the discussion of the paper, avoidant actors’ withdrawal may only influence partners’ power if partners detect such behavior. Moreover, providing support that partner’s perceptions of withdrawal captured actual behavioral dynamics, the links between actors’ avoidance and partners’ power via actors’ withdrawal was supported by observational coding of withdrawal (see Study 4 Results in paper).

**Table S8**

*Descriptive Statistics and Cronbach’s Alphas for Self-Reported Withdrawal*

|  | Women | | |  | Men | | |  |  |  |  |
| --- | --- | --- | --- | --- | --- | --- | --- | --- | --- | --- | --- |
| Variable | *M* | *SD* | α |  | *M* | *SD* | α |  | *t* | *\|d*\| |  |
| Self-Reported Withdrawal | 3.46 | 1.77 | .82 |  | 3.12 | 1.68 | .80 |  | -1.61 | 0.14 |  |

*Note.* *t* = Results of paired samples *t* tests. *N* = 138 couples.

**p* < .05. ***p* < .01. ****p* < .001 (two-tailed).

**Table S9**

*Results of APIMeM Analyses Estimating the Actor and Partner Associations between Attachment Avoidance and Power With Self-Reported Withdrawal as Mediator*

|  |  | | Predictor: Avoidance | | | | |  |
| --- | --- | --- | --- | --- | --- | --- | --- | --- |
|  |  | *b* | | 95% CI | *SE* | *p* | \|Δ\| | |
| Direct effects |  |  | |  |  |  |  | |
| Avoidance 🡪 Withdrawal |  |  | |  |  |  |  | |
| Actor (a) |  | **0.36** | | [0.15, 0.58] | 0.11 | .001 | 0.20^W^  0.21^M^ | |
| Partner (p_1_) |  | -0.03 | | [-0.24, 0.18] | 0.11 | .806 | 0.02^W^  0.02^M^ | |
| Withdrawal 🡪 Power |  |  | |  |  |  |  | |
| Actor (b) |  | -0.06 | | [-0.13, 0.01] | 0.04 | .094 | 0.07^W^  0.06^M^ | |
| Partner (p_3_) |  | -0.04 | | [-0.11, 0.03] | 0.03 | .287 | 0.04^W^  0.04^M^ | |
| Avoidance 🡪 Power |  |  | |  |  |  |  | |
| Actor (c’) |  | **-0.12** | | [-0.24, -0.01] | 0.06 | .042 | 0.13^W^  0.12^M^ | |
| Partner (p_2_) |  | **-0.14** | | [-0.28, -0.00] | 0.07 | .047 | 0.16^W^  0.14^M^ | |
| Indirect partner effects |  |  | |  |  |  |  | |
| Total |  | **-0.15** | | [-0.29, -0.02] | 0.07 | .029 |  | |
| Total indirect |  | -0.01 | | [-0.05, 0.01] | 0.02 | .431 |  | |
| Specific indirect |  | -0.01 | | [-0.05, 0.01] | 0.01 | .338 |  | |

*Note.* *b* = Unstandardized regression coefficient, CI = Bootstrapped 95% confidence interval, Δ = Effect size, W = women, M = men. Effects were pooled across women and men unless a model with different effects for women and men was preferred according to Likelihood-Ratio tests (indicated by the use of superscripts in columns *b* to *p*). *N* = 138 couples. The bold values indicate significant *b* coefficients or significant indirect effects (CI does not include zero). The specific indirect effects test if actor’s self-reported withdrawal is a mediator in the link between actor’s avoidance and partner’s power.

## Results for Attachment Anxiety (Study 4)

The aim of Study 4 focused on the links between attachment avoidance and partners’ power. We present analogous APIMeM analyses for attachment anxiety as supplemental analyses. Descriptive statistics and gender differences appear in Table S10, and correlations across measures in Table S11.

In the first APIMeM with perceived partner withdrawal (equivalent to Test 1 in the paper), actor’s attachment anxiety was negatively linked to partner’s perception of actor’s withdrawal, which in turn was negatively linked to partner’s power (see Table S12). Thus, anxious individuals are perceived as low in withdrawal behavior by their partners, which relates to partner’s feeling greater relationship power. Moreover, attachment anxiety was positively linked to perceiving the partner as high in withdrawal. Thus, the results for attachment anxiety differed from those of attachment avoidance.

In the second APIMeM based on the conflict discussion measures (Test 2 in the paper) only one significant effect was found: Greater withdrawal behavior was associated with lower partner’s power (see Table S12). This is in line with the results theorized and reported in the paper: Distancing behaviors limit partner’s power.

**Table S10**

*Descriptive Statistics and Cronbach’s Alphas for Attachment Anxiety*

|  | Women | | |  | Men | | |  |  |  |  |
| --- | --- | --- | --- | --- | --- | --- | --- | --- | --- | --- | --- |
| Variable | *M* | *SD* | α |  | *M* | *SD* | α |  | *t* | *\|d*\| |  |
| Anxiety | 3.24 | 1.15 | .82 |  | 2.86 | 1.03 | .79 |  | -3.23** | 0.28 |  |

*Note.* *t* = Results of paired samples *t* tests. *N* = 138 couples.

**p* < .05. ***p* < .01. ****p* < .001 (two-tailed).

**Table S11**

*Zero-Order Correlations Across all Attachment, Withdrawal, and Power Measures*

| Variable |  | Avoidance | Anxiety | Perceived Withdrawal | Withdrawal Conflict | Self-reported Withdrawal | Power | Power Conflict |
| --- | --- | --- | --- | --- | --- | --- | --- | --- |
| Avoidance |  | .11 | .18* | -.10 | -.09 | .08 | -.17* | -.03 |
| Anxiety |  | .20* | .15 | .15 | -.07 | .15 | -.33*** | -.05 |
| Perceived Withdrawal |  | .17* | .26** | .81*** | -.05 | -.05 | -.06 | -.07 |
| Withdrawal Conflict |  | -.13 | .02 | -.15 | .01 | .10 | .01 | -.04 |
| Self-reported Withdrawal |  | .31*** | .08 | .23** | -.03 | -.06 | -.10 | .08 |
| Power |  | -.16 | -.25** | -.33*** | -.09 | -.17* | .19* | .18* |
| Power Conflict |  | -.02 | -.06 | -.08 | .02 | -.03 | .10 | .17 |

*Note.* Correlations within actors (e.g., actors’ avoidance and actors’ experienced power) are presented separately for women (above the diagonal) and men (below the diagonal). Correlations across partners (e.g., women’s and men’s avoidance) are presented in the diagonal. *N* = 138 couples.

Avoidance, anxiety, self-reported withdrawal, perception of partner’s withdrawal, and power were general self-report measures. Withdrawal during conflict discussion was rated by independent observers. Power during conflict discussion was a situational self-report measure.

**p* < .05. ***p* < .01. ****p* < .001 (two-tailed).

**Table S12**

*Results of APIMeM Analyses Estimating the Actor and Partner Associations between Attachment Anxiety and Power (Left: Generalized Power; Right: Power During Conflict Discussion) With Withdrawal Behavior (Left: Perceptions of Partner’s General Withdrawal; Right: Observer Rated Withdrawal During Conflict Discussion) as Mediator*

|  | Questionnaire Measures | | | | |  |  | |  | | | Conflict Measures | | | | |
| --- | --- | --- | --- | --- | --- | --- | --- | --- | --- | --- | --- | --- | --- | --- | --- | --- |
|  | *b* | 95% CI | *SE* | *p* | \|Δ\| | | |  | | *b* | 95% CI | | *SE* | *p* | \|Δ\| |  |
| Direct effects |  |  |  |  |  | | |  | |  |  | |  |  |  |  |
| Anxiety 🡪 Withdrawal |  |  |  |  |  | | |  | |  |  | |  |  |  |  |
| Actor (a) | **0.45** | [0.26, 0.64] | 0.10 | <.001 | 0.25^W^  0.27^M^ | | |  | | -0.02 | [-0.06, 0.03] | | 0.03 | .502 | 0.04^W^  0.03^M^ |  |
| Partner (p_1_) | **-0.20** | [-0.39, -0.02] | 0.09 | .029 | 0.11^W^  0.12^M^ | | |  | | 0.05 | [-0.01, 0.13] | | 0.04 | .147 | 0.11^W^  0.05^M^ |  |
| Withdrawal 🡪 Power |  |  |  |  |  | | |  | |  |  | |  |  |  |  |
| Actor (b) | **-0.11** | [-0.18, -0.05] | 0.03 | .001 | 0.12^W^  0.11^M^ | | |  | | -0.08 | [-0.37, 0.17] | | 0.14 | .592 | 0.09^W^  0.08^M^ |  |
| Partner (p_3_) | -0.03 | [-0.09, 0.03] | 0.03 | .279 | 0.03^W^  0.03^M^ | | |  | | **-0.25** | [-0.46, -0.02] | | 0.11 | .025 | 0.28^W^  0.25^M^ |  |
| Anxiety 🡪 Power |  |  |  |  |  | | |  | |  |  | |  |  |  |  |
| Actor (c’) | **-0.21** | [-0.30, -0.12] | 0.05 | <.001 | 0.24^W^  0.21^M^ | | |  | | -0.05 | [-0.20, 0.11] | | 0.08 | .542 | 0.06^W^  0.05^M^ |  |
| Partner (p_2_) | -0.00 | [-0.10, 0.10] | 0.05 | .984 | 0.00^W^  0.00^M^ | | |  | | -0.04 | [-0.19, 0.12] | | 0.08 | .630 | 0.04^W^  0.04^M^ |  |
| Indirect partner effects |  |  |  |  |  | | |  | |  |  | |  |  |  |  |
| Total | 0.01 | [-0.09, 0.10] | 0.05 | .894 |  | | |  | | -0.04 | [-0.19, 0.13] | | 0.08 | .631 |  |  |
| Total indirect | 0.01 | [-0.03, 0.05] | 0.02 | .687 |  | | |  | | 0.00 | [-0.03, 0.02] | | 0.01 | .987 |  |  |
| Specific indirect | **0.02** | [0.00, 0.06] | 0.01 | .087 |  | | |  | | 0.00 | [-0.01, 0.02] | | 0.01 | .549 |  |  |

*Note.* *b* = Unstandardized regression coefficient, CI = Bootstrapped 99% confidence interval, Δ = Effect size, W = women, M = men. Effects were pooled across women and men unless a model with different effects for women and men was preferred according to Likelihood-Ratio tests (indicated by the use of superscripts in columns *b* to *p*). *N* = 138 couples. The bold values indicate significant *b* coefficients or significant indirect effects (CI does not include zero). The specific indirect effects test (a) if partner’s perception of actor’s withdrawal is a mediator in the link between actor’s anxiety and partner’s power, and (b) if observed withdrawal behavior of an actor is a mediator in the link between actor’s anxiety and partner’s power.

# Additional Mediation Results (Study 5: Dyadic Daily Diary)

## Descriptive Statistics and Reliabilities of all Variables (Study 5)

**Table S13**

*Descriptive Statistics, Cronbach’s Alphas, Tests of Gender Differences, and Zero-Order Correlations Across Measures Based on Aggregated Means on Person Level*

|  | Women | | |  | Men | | |  |  |  |  | Correlations | | | | |  |
| --- | --- | --- | --- | --- | --- | --- | --- | --- | --- | --- | --- | --- | --- | --- | --- | --- | --- |
| Variable | *M* | *SD* | α |  | *M* | *SD* | α |  | *t* | *\|d*\| |  | 1. | 2. | 3. | 4. | 5. | |
| 1. Avoidance | 3.02 | 1.06 | .76 |  | 2.79 | 0.90 | .72 |  | 2.08* | 0.17 |  | .09 | .17* | .25** | .34*** | -.16 | |
| 2. Anxiety | 3.28 | 1.09 | .83 |  | 2.80 | 0.98 | .82 |  | 4.20*** | 0.34 |  | .22** | -.11 | .31*** | .28*** | -.18* | |
| 3. Perceived Partner Withdrawal | 2.03 | 0.95 | .94 |  | 1.91 | 0.81 | .94 |  | 1.88 | 0.15 |  | .18* | .16* | .56*** | .88*** | -.46*** | |
| 4. Self-Rated Withdrawal | 1.97 | 0.91 | .94 |  | 2.06 | 0.94 | .95 |  | -1.18 | 0.10 |  | .33*** | .10 | .81*** | .39*** | -.41*** | |
| 5. Power | 5.66 | 1.08 | .95 |  | 5.50 | 1.06 | .95 |  | 1.49 | 0.12 |  | -.11 | -.14 | -.40*** | -.32*** | .27*** | |

*Note.* Reliability of the daily measures (3.-5.) was computed as within-person reliability (i.e., consistency across days). *t* = Results of paired samples *t* tests. Correlations within actors (e.g., actors’ avoidance and actors’ experienced power) are presented separately for women (above the diagonal) and men (below the diagonal). Correlations across partners (e.g., women’s and men’s avoidance) are presented in the diagonal. *N* = 151 couples.

**p* < .05. ***p* < .01. ****p* < .001 (two-tailed).

## Results of Model Fit Statistics Examining Gender as a Moderator (Study 5)

**Table S14**

*Results of Model Fit Statistics Examining Gender as a Moderator Based on Deviance Information Criterion (DIC)*

|  |  | Saturated Model | Constrained Model |
| --- | --- | --- | --- |
| Model 1 | Predictor: Avoidance  Mediator: Perceived Withdrawal  Outcome: Power | 36728 | 36712 |
|  |  |  |  |
| Model 2 | Predictor: Anxiety  Mediator: Perceived Withdrawal  Outcome: Power | 36766 | 37649 |
|  |  |  |  |
| Model 3 | Predictor: Avoidance  Mediator: Withdrawal Self  Outcome: Power | 19474 | 19464 |
|  |  |  |  |

*Note.* Lower DIC values indicate better model fit (similar to AIC). Saturated model: All effects are freely estimated. Constrained model: Actor and partner effects are set equal for women and men.

## Results Examining Avoidance as a Predictor of Perceived Withdrawal (Mediator) and Power (Outcome) For Separate Subsamples (Study 5)

The data of Study 5 came from two independent samples, which differed slightly in their power measure (see section “Scales and Items Used to Assess Attachment, Power, Withdrawal, and Control Variables (Studies 1-5)”). APIMeM results for the two different samples show that the focal indirect test is significant and similar across the two samples (see Tables S16). Thus, the results replicated across the samples using slightly different worded assessments of daily power, justifying aggregating the two samples to maximize statistical power as done in the paper.

**Table S15**

*Study 5: –Results of Multilevel APIMeM Analyses Estimating the Actor and Partner Associations between Avoidance, Withdrawal, and Power for Sample 1 and 2*

|  | *Estimate* | 95% CI | *p* |
| --- | --- | --- | --- |
| **SAMPLE 1** |  |  |  |
| Within level |  |  |  |
| Withdrawal 🡪 Power |  |  |  |
| Actor (b_w_) | **-0.29** | [-0.33, -0.26] | < .001 |
| Partner (p_w3_) | **-0.08** | [-0.12, -0.04] | < .001 |
|  |  |  |  |
| Between level |  |  |  |
| Avoidance 🡪 Withdrawal |  |  |  |
| Actor (a) | **0.17** | [0.05, 0.33] | < .001 |
| Partner (p_1_) | **0.16** | [0.04, 0.29] | < .001 |
| Withdrawal 🡪 Power |  |  |  |
| Actor (b) | **-0.59** | [-0.76, -0.35] | < .001 |
| Partner (p_3_) | -0.01 | [-0.25, 0.22] | .940 |
| Avoidance 🡪 Power |  |  |  |
| Actor (c’) | -0.03 | [-0.16, 0.12] | .680 |
| Partner (p_2_) | 0.00 | [-0.12, 0.14] | .999 |
|  |  |  |  |
| Indirect effect |  |  |  |
| Avoidance 🡪 Partner withdrawal 🡪 Partner power (p_1_*b) | **-0.09** | [-0.162, -0.024] | < .001 |
|  |  |  |  |
| **SAMPLE 2** |  |  |  |
| Within level |  |  |  |
| Withdrawal 🡪 Power |  |  |  |
| Actor (b_w_) | 0.02 | [-0.03, 0.06] | .440 |
| Partner (p_w3_) | 0.01 | [-0.03, 0.06] | .670 |
|  |  |  |  |
| Between level |  |  |  |
| Avoidance 🡪 Withdrawal |  |  |  |
| Actor (a) | **0.17** | [0.01, 0.32] | .030 |
| Partner (p_1_) | **0.19** | [0.03, 0.33] | < .001 |
| Withdrawal 🡪 Power |  |  |  |
| Actor (b) | **-0.51** | [-0.78, -0.26] | < .001 |
| Partner (p_3_) | -0.02 | [-0.31, 0.28] | .900 |
| Avoidance 🡪 Power |  |  |  |
| Actor (c’) | -0.09 | [-0.28, -0.09] | .410 |
| Partner (p_2_) | **0.25** | [0.01, 0.45] | .040 |
|  |  |  |  |
| Indirect effect |  |  |  |
| Avoidance 🡪 Partner withdrawal 🡪 Partner power (p_1_*b) | **-0.09** | [-0.192, -0.019] | < .001 |

*Note.* CI = 95% credibility interval. Effects were pooled across women and men. *N* = 78 couples. The bold values indicate significant estimates or significant indirect effects (CI does not include zero).

## Results Examining Anxiety as a Predictor of Perceived Withdrawal (Mediator) and Power (Outcome) (Study 5)

The aim of Study 5 focused on the links between attachment avoidance and partners’ power. We present analogous APIMeM analyses for attachment anxiety as supplemental analyses. Actors’ attachment anxiety was positively related to actors perceiving partners high in withdrawal. Perceiving partners high in withdrawal was negatively linked to power for actors. There was no indirect effect between attachment anxiety, partner perceived withdrawal, and partner’s power (see Table S16).

**Table S16**

*Results of Multilevel APIMeM Analyses Estimating the Actor and Partner Associations between Anxiety, Withdrawal, and Power*

|  | *Estimate* | 95% CI | *p* |
| --- | --- | --- | --- |
| Within level |  |  |  |
| Withdrawal 🡪 Power |  |  |  |
| Actor (b_w_) | **-0.17** | [-0.20, -0.14] | < .001 |
| Partner (p_w3_) | **-0.05** | [-0.08, -0.02] | < .001 |
|  |  |  |  |
| Between level |  |  |  |
| Anxiety 🡪 Withdrawal |  |  |  |
| Actor (a) | **0.18** | [0.09, 0.29] | < .001 |
| Partner (p_1_) | 0.06 | [-0.04, 0.15] | .160 |
| Withdrawal 🡪 Power |  |  |  |
| Actor (b) | **-0.53** | [-0.69, -0.34] | < .001 |
| Partner (p_3_) | 0.00 | [-0.21, 0.20] | .920 |
| Anxiety 🡪 Power |  |  |  |
| Actor (c’) | -0.05 | [-0.16, 0.04] | .360 |
| Partner (p_2_) | 0.06 | [-0.08, 0.17] | .280 |
|  |  |  |  |
| Indirect effect |  |  |  |
| Anxiety 🡪 Partner withdrawal 🡪 Partner power (p_1_*b) | -0.03 | [-0.080, 0.020] | .0160 |

*Note.* CI = 95% credibility interval. Effects were pooled across women and men. *N* = 151 couples. The bold values indicate significant estimates or significant indirect effects (CI does not include zero).

## Results Examining Avoidance as a Predictor of Self-Reported Withdrawal (Mediator) and Power (Outcome) (Study 5)

In Study 5, self-rated withdrawal was assessed with two items. See section “Scales and Items Used to Assess Attachment, Power, Withdrawal, and Control Variables (Studies 1-5)”. Both items were highly correlated for women and men when aggregated across days (*r*s = .75/.83, *p*s < .001).

**Table S17**

*Results of Multilevel APIMeM Analyses Estimating the Actor and Partner Associations between Avoidance, Withdrawal, and Power*

|  | *Estimate* | 95% CI | *p* |
| --- | --- | --- | --- |
| Within level |  |  |  |
| Withdrawal 🡪 Power |  |  |  |
| Actor (b_w_) | **-0.23** | [-0.27, 0.18] | < .001 |
| Partner (p_w3_) | **-0.13** | [-0.17, -0.09] | < .001 |
|  |  |  |  |
| Between level |  |  |  |
| Avoidance 🡪 Withdrawal |  |  |  |
| Actor (a) | **0.34** | [0.22, 0.48] | < .001 |
| Partner (p_1_) | -0.02 | [-0.20, -0.11] | .780 |
| Withdrawal 🡪 Power |  |  |  |
| Actor (b) | **-0.35** | [-0.57, -0.18] | < .001 |
| Partner (p_3_) | -0.15 | [-0.33, 0.05] | .220 |
| Avoidance 🡪 Power |  |  |  |
| Actor (c’) | -0.01 | [-0.15, 0.13] | .860 |
| Partner (p_2_) | -0.05 | [-0.18, 0.10] | .660 |
|  |  |  |  |
| Indirect effect |  |  |  |
| Avoidance 🡪 Withdrawal 🡪 Partner power (p_1_*b) | -0.05 | [-0.12, 0.03] | .220 |

*Note.* CI = 95% credibility interval. Effects were pooled across women and men. *N* = 151 couples. The bold values indicate significant estimates or significant indirect effects (CI does not include zero).

The results show that avoidance is positively linked to actor’s withdrawal. However actors’ withdrawal was not linked to partners’ power, and the indirect effect was not significant. Thus, as we expected and reiterated in the discussion of the paper, avoidant actors’ withdrawal likely only shapes partners’ power if partners detect withdrawal (see General Discussion in paper for further discussion).

## Results Examining Avoidance as a Predictor of Perceived Withdrawal (Mediator) and Power (Outcome) Using Dynamic Structural Equation Modelling to Analyze Lagged Effects on the Within-Level (Study 5)

Our primary analyses in Study 5 focused on within-day processes for theoretical reasons. We expect that withdrawal immediately undercuts power within specific interactions, such as conflict interactions assessed in the lab (as in Study 4) and interactions that occur within days (as modelled in Study 5). These within-interaction processes should produce average patterns that account for why between-person differences in attachment avoidance produce between-person differences in partner’s perceived withdrawal and power. However, these should be within-interaction or within-day effects accumulating across days rather than building from lagged- or time-delayed effects of withdrawal.

Nonetheless, as encouraged during the review process, we conducted lagged analyses to examine whether today’s perceived partner withdrawal predicts tomorrow’s power (and vice versa) to assess whether across-day links supported the causal direction of our theorizing. We used dynamic structural equation modeling (D-SEM; Asparouhov et al., 2018), which combined our multilevel SEM analyses with a time-series approach that accounts for lagged relations between variables. We extended the model described in the manuscript (Study 5, Analytical Strategy) by incorporating lagged variables for both power and perceived partner withdrawal. We modelled autoregressive effects by including paths from each variable’s value at time *t* to its value at time *t* +1, thereby accounting for the temporal stability of each construct. The across-day links were modelled by specifying reciprocal cross-lagged effects; that is, we simultaneously predicted tomorrow’s power from today’s partner-perceived withdrawal and predicted tomorrow’s partner-perceived withdrawal from today’s power. This dual specification allowed us to examine the bidirectional influence of these variables over time while controlling for their stability.

The results are displayed in Table S18. On the between-level, the results were very similar to those reported in the manuscript (Table 9). On the within-level, the within-day results were also very similar. No significant lagged effects were found. Thus, despite within-person linkages on the same day, today’s power did not significantly predict tomorrow’s perceived partner withdrawal and nor did today’s perceived partner withdrawal significantly predict tomorrow’s power.

**Table S18**

*Results of D-SEM Analyses Estimating the Actor and Partner Associations between Avoidance, Perceived Partner Withdrawal, and Power*

|  | *Estimate* | 95% CI | *p* |
| --- | --- | --- | --- |
| Within level |  |  |  |
| Perceived Partner Withdrawal 🡪 Power |  |  |  |
| Actor (b_w_) | **-0.17** | [-0.19, -0.14] | < .001 |
| Partner (p_w3_) | **-0.05** | [-0.08, -0.02] | < .001 |
|  |  |  |  |
| Lagged Effects |  |  |  |
| Perceived Partner Withdrawal (Day t) 🡪 Power (Day t +1) |  |  |  |
| Actor (b_wlag_) | 0.00 | [-0.04, 0.03] | .916 |
| Partner (p_w3lag_) | 0.02 | [-0.01, 0.05] | .192 |
|  |  |  |  |
| Power (Day t) 🡪 Perceived Partner Withdrawal (Day t +1) |  |  |  |
| Actor (b_wreverselag_) | -0.02 | [-0.05, 0.00] | .110 |
| Partner (p_w3reverselag_) | 0.00 | [-0.03, 0.03] | .970 |
|  |  |  |  |
| Between level |  |  |  |
| Avoidance 🡪 Perceived Partner Withdrawal |  |  |  |
| Actor (a) | **0.18** | [0.08, 0.27] | < .001 |
| Partner (p_1_) | **0.16** | [0.07, 0.26] | .004 |
| Perceived Partner Withdrawal 🡪 Power |  |  |  |
| Actor (b) | **-0.59** | [-0.76, -0.41] | < .001 |
| Partner (p_3_) | 0.02 | [-0.18, 0.19] | .840 |
| Avoidance 🡪 Power |  |  |  |
| Actor (c’) | -0.04 | [-0.16, 0.08] | .492 |
| Partner (p_2_) | 0.10 | [-0.03, 0.22] | .104 |
|  |  |  |  |
| Indirect effect |  |  |  |
| Avoidance 🡪 Perceived partner Withdrawal 🡪 Partner power (p_1_*b) | **-0.09** | [-0.172, -0.031] | .004 |

*Note.* CI = 95% credibility interval. Effects were pooled across women and men. *N* = 151 couples. The bold values indicate significant estimates or significant indirect effects (CI does not include zero).

# References

Asparouhov, T., Hamaker, E. L., & Muthén, B. (2018). Dynamic structural equation models. *Structural Equation Modeling, 25*(3), 359–388.

Baranger, D. A., Finsaas, M. C., Goldstein, B. L., Vize, C. E., Lynam, D. R., & Olino, T. M. (2023). Tutorial: Power analyses for interaction effects in cross-sectional regressions. *Advances in Methods and Practices in Psychological Science, 6*(3), 25152459231187531.

Hayes, A. F. (2017). *Introduction to mediation, moderation, and conditional process analysis: A regression-based approach.* Guilford publications.

Körner, R., & Schütz, A. (2024). Power balance and relationship quality: An overstated link. *Social Psychological and Personality Science.* Advance online publication.

MacKinnon, D. P., Lockwood, C. M., & Williams, J. (2004). Confidence limits for the indirect effect: Distribution of the product and resampling methods. *Multivariate Behavioral Research, 39*(1), 99–128.

Murphy, B. A., Casto, K. V., Watts, A. L., Costello, T. H., Jolink, T. A., Verona, E., & Algoe, S. B. (2022). “Feeling Powerful” versus “Desiring Power”: A pervasive and problematic conflation in personality assessment? *Journal of Research in Personality, 101,* Article 104305.

Overall, N. C., Maner, J. K., Hammond, M. D., Cross, E. J., Chang, V. T., Low, R. S. T., Girme, Y. U., Jayamaha, S. D., Reid, C. J., & Sasaki, E. (2023). Actor and partner power are distinct and have differential effects on social behavior. *Journal of Personality and Social Psychology, 124*(2), 311–343.

Preacher, K. J., & Hayes, A. F. (2008). Asymptotic and resampling strategies for assessing and comparing indirect effects in multiple mediator models. *Behavior Research Methods, 40*(3), 879–891.

Traeder, C. K., & Zeigler-Hill, V. (2020). The desire for power and perceptions of heterosexual romantic relationships: The moderating roles of perceived power and gender. *Sex Roles, 82,* 66–80.

Vize, C.E., Baranger, D.A.A., Finsaas, M. C., Goldstein, B. L., Olino, T., & Lynam, D. R. (2023). Moderation effects in personality disorder research. *Personality Disorders: Theory, Research, and Treatment, 14,* 118–126.
